# Screening Signals of Reference-Defined Metabolic Syndrome Using HbA1c and LDL Cholesterol: An Explainable Machine Learning Study

**DOI:** 10.3390/diagnostics16183048

**Published:** 2026-09-20

**Authors:** Osman Demir

**Affiliations:** Department of Biostatistics, Faculty of Medicine, Tokat Gaziosmanpaşa University, Tokat 60100, Türkiye; osmand.demir@gop.edu.tr

**Keywords:** metabolic syndrome, screening, HbA1c, LDL cholesterol, machine learning, gradient boosting, calibration, decision curve analysis, SHAP, incorporation bias

## Abstract

**Background:** Metabolic syndrome (MetS) is characterized by the clustering of central adiposity, elevated blood pressure, dysglycemia, and atherogenic dyslipidemia. Machine learning models for metabolic syndrome may show inflated performance when predictors overlap with the diagnostic criteria used to define the reference outcome. Glucose, triglycerides, and high-density lipoprotein (HDL) cholesterol are components of the National Cholesterol Education Program Adult Treatment Panel III (NCEP ATP III) definition of MetS; therefore, their use as predictors may introduce incorporation bias. This study aimed to evaluate whether glycated hemoglobin A1c (HbA1c) and low-density lipoprotein (LDL) cholesterol provide screening information for reference-defined MetS and to quantify the effect of predictor–outcome overlap. **Methods:** This retrospective cross-sectional analysis used de-identified routine-care data comprising 17,981 laboratory records, including 8982 records with MetS and 8999 without MetS. MetS was defined according to the updated NCEP ATP III criteria, with all five components available for reference classification from the same clinical encounter. The primary model used HbA1c and LDL cholesterol. For comparison, two additional models were evaluated: a criterion-component model using glucose, triglycerides, and HDL cholesterol, and a full biochemical model using all five variables. Gradient boosting was evaluated within a patient-level development and held-out internal test framework, with no patient shared between partitions. Model performance was assessed using ROC-AUC, threshold-based classification metrics, Brier score, calibration, decision curve analysis, and SHAP-based explainability. **Results:** The primary HbA1c–LDL cholesterol model showed moderate-to-strong discrimination for reference-defined MetS, with an ROC-AUC of 0.810, accuracy of 0.733, sensitivity of 0.824, specificity of 0.641, F1-score of 0.756, and Brier score of 0.174. The criterion-component model using glucose, triglycerides, and HDL cholesterol achieved a higher ROC-AUC of 0.935, consistent with a strong influence of predictor–outcome overlap. The full biochemical model achieved the highest ROC-AUC of 0.956 and Brier score of 0.084; however, this performance should be interpreted as an upper-bound estimate influenced by incorporation bias. SHAP analysis of the primary HbA1c–LDL cholesterol model indicated that HbA1c contributed more strongly than LDL cholesterol to the model output. **Conclusions:** HbA1c and LDL cholesterol provided measurable biochemical screening information for reference-defined MetS, although their discriminatory performance was lower than models including diagnostic criterion components. The substantially higher performance of models containing glucose, triglycerides, and HDL cholesterol is consistent with a substantial influence of incorporation bias when diagnostic criteria components are used as predictors. These findings should be interpreted as hypothesis-generating and limited to single-center internal validation. External, temporal, multicenter, and prospective validation is required before any clinical application can be considered.

## 1. Introduction

Metabolic syndrome (MetS) is a multifactorial clinical disorder characterized by the concurrent occurrence of several cardiometabolic abnormalities, including impaired glucose regulation, dyslipidemia, elevated blood pressure, central adiposity, and insulin resistance [1]. Its definition and clinical presentation may vary across diagnostic frameworks and populations, reflecting the heterogeneous nature of the syndrome [2,3]. MetS is widely recognized as a major public health issue because of its strong association with cardiovascular disease, type 2 diabetes mellitus, stroke, and increased mortality risk [4]. Because MetS typically progresses gradually and may remain clinically silent in its early stages, timely identification of individuals at risk is essential to enable effective preventive healthcare interventions [5,6].

The diagnosis of MetS is traditionally based on predefined clinical and biochemical criteria, including elevated fasting glucose, increased triglyceride levels, reduced high-density lipoprotein (HDL) cholesterol, elevated blood pressure, and increased waist circumference (WC) [7]. According to the National Cholesterol Education Program Adult Treatment Panel III (NCEP ATP III) criteria, MetS is defined by the presence of at least three of the following five components: abdominal obesity, elevated triglycerides, reduced high-density lipoprotein (HDL) cholesterol, elevated blood pressure, and elevated fasting glucose [7]. Although these criteria are useful in routine clinical practice, conventional threshold-based definitions may not fully capture the continuous relationships and heterogeneity underlying metabolic risk [8,9]. Individuals who do not meet the required threshold of at least three diagnostic components may exhibit early biochemical alterations indicative of metabolic dysregulation. Therefore, data-driven methods capable of evaluating screening signals for reference-defined MetS using routinely available clinical information may be useful [1,10].

Routine biochemical parameters are an accessible, low-cost, and widely used source of information for assessing cardiometabolic risk. These parameters have also been used in previous machine learning-based MetS prediction studies [1]. Among routine laboratory markers, glycated hemoglobin A1c (HbA1c) reflects long-term glycemic exposure and has been examined in relation to MetS [11]. LDL cholesterol was included not as a diagnostic component of MetS, but as a routinely measured marker of lipid metabolism that may provide complementary screening information beyond the biochemical components embedded in the NCEP ATP III definition. Glucose, triglycerides, HDL cholesterol, and LDL cholesterol capture distinct aspects of glucose metabolism and lipid homeostasis and have recognized biochemical relevance in MetS [12]. These biomarkers are typically obtained during standard health evaluations and may provide valuable discriminatory information for classifying MetS status, as defined by the NCEP ATP III criteria [7,13]. Consequently, developing classification models based on routinely measured biochemical variables may offer practical advantages, particularly in settings where detailed anthropometric or clinical assessments are not consistently available. Previous machine learning studies have supported the feasibility of data-driven models in MetS and related metabolic disorders [1,13]. In addition, anthropometric indicators remain important complementary clinical measures for cardiometabolic risk assessment [14].

Machine learning techniques are increasingly used in clinical prediction research because they can represent complex structures, nonlinear effects, and interactions between predictors. In contrast to traditional statistical approaches, such algorithms may yield better classification accuracy when associations between predictors and outcomes deviate from linearity [13,15]. This is especially pertinent for MetS and related cardiometabolic diseases, which result from disturbances across multiple metabolic pathways rather than a single isolated defect [12,16]. Consequently, models such as random forest, gradient boosting, XGBoost, and neural network-based architectures may be well-suited to uncover latent patterns within routinely collected biochemical data [15]. Previous machine learning studies in MetS and related cardiometabolic disorders further support the use of such data-driven approaches in this context [1,13]. The algorithms evaluated in this study represent different modelling principles. Logistic regression provides a conventional linear statistical reference model and estimates additive associations between predictors and outcome. Naïve Bayes is a probabilistic classifier based on conditional independence assumptions. K-nearest neighbors classifies observations according to the labels of nearby cases in the predictor space, whereas support vector machines define separating margins between classes, often using kernel functions to handle nonlinear boundaries. Decision trees partition the predictor space through sequential threshold-based rules and are easy to interpret but may be unstable when used alone. Random forests improve stability by averaging many decision trees trained on bootstrap samples and random subsets of predictors. Gradient boosting also builds an ensemble of decision trees, but does so sequentially, with each new tree focusing on the errors made by previous trees [17]. XGBoost is an optimized implementation of gradient boosting that includes additional regularization and computational improvements [18]. Multilayer perceptrons are neural network models that learn nonlinear patterns through hidden layers. This range of algorithms allowed us to compare conventional, probabilistic, instance-based, margin-based, tree-based, boosting-based, and neural network approaches within the same development-set validation framework [15].

Despite their potential advantages, machine learning models should not be judged solely based on their overall accuracy. In clinical classification tasks, multiple performance metrics, including sensitivity, specificity, precision, F1-score, and area under the receiver operating characteristic curve (ROC-AUC), are required to provide a comprehensive assessment of model performance [19,20]. In addition, cross-validation procedures are essential for evaluating model stability and mitigating the risk of overfitting [21,22]. A comparative evaluation of different machine learning algorithms within a consistent validation framework can help identify the most appropriate model for classifying MetS [1,15].

Another key issue is model interpretability. Although complex algorithms may deliver strong classification results, their value in clinical settings can be restricted if the logic behind their predictions is unclear [23,24]. Approaches from explainable artificial intelligence can help overcome this challenge by quantifying the contribution of each feature to the model’s output [25,26]. Among these, SHAP-based methods offer both global and case-specific explanations of predictions and can elucidate how biochemical variables, including glucose, glycated hemoglobin, triglycerides, high-density lipoprotein cholesterol, and low-density lipoprotein cholesterol, affect MetS classification [25,27]. Therefore, incorporating SHAP analysis into the modelling workflow may enhance the transparency and interpretability of screening-oriented ML models [27,28].

In this study, we evaluated whether HbA1c and LDL cholesterol provide screening information for reference-defined MetS and examined how model performance changes when biochemical components of the MetS definition are included as predictors. The primary model used HbA1c and LDL cholesterol only. Two comparison models were used to quantify predictor–outcome overlap: a criterion-component model including glucose, triglycerides, and HDL cholesterol, and a full biochemical model including all five variables. For contextual methodological comparison, nine supervised learning algorithms were evaluated: logistic regression, k-nearest neighbors, support vector machine, decision tree, random forest, gradient boosting, XGBoost, Gaussian naïve Bayes, and multilayer perceptron. Model performance was assessed using development-set cross-validation, held-out internal test-set evaluation, calibration analysis, decision curve analysis, threshold-based performance metrics, and multiple classification metrics. In addition, the primary HbA1c–LDL cholesterol gradient-boosting model was interpreted using SHAP-based explainability methods to clarify how HbA1c and LDL cholesterol contributed to the model output.

A key methodological challenge in laboratory-based MetS modelling is the overlap between predictor variables and the diagnostic criteria used to define the reference outcome. In particular, glucose, triglycerides, and HDL cholesterol are components of the NCEP ATP III definition and may therefore introduce incorporation bias when used as model inputs. Previous machine learning studies on MetS have often focused primarily on overall discrimination, whereas fewer studies have explicitly examined predictor–outcome overlap, calibration, exploratory clinical utility, and model explainability within the same analytical framework.

The present study addresses this gap by evaluating HbA1c and LDL cholesterol as primary screening markers for reference-defined MetS while using criterion-component and full biochemical models as comparison analyses to quantify the influence of predictor–outcome overlap. This design allows the primary model to be interpreted not as an independent etiological risk-prediction tool or stand-alone diagnostic procedure, but as an internally validated, hypothesis-generating screening model.

From a clinical informatics perspective, this study proposes an explainable, laboratory-based screening framework using routinely available HbA1c and LDL cholesterol measurements. Accordingly, the contribution of this study extends beyond algorithm comparison to include explicit evaluation of incorporation bias, model calibration, exploratory decision-curve analysis, and SHAP-based model transparency.

The remainder of this paper is organized as follows. Section 2 describes the study design, outcome definition, biochemical feature sets, preprocessing strategy, model development, validation framework, performance evaluation, calibration, decision curve analysis, and SHAP-based explainability approach. Section 3 presents the descriptive characteristics of the dataset, primary screening model performance, feature-set comparisons, contextual algorithm benchmarking, reference-model comparisons, calibration findings, decision curve analysis, confusion matrix, and interpretability results. Section 4 interprets the main findings, methodological implications, limitations, and directions for future validation.

## 2. Materials and Methods

### 2.1. Study Design and Dataset

**Figure 1:**
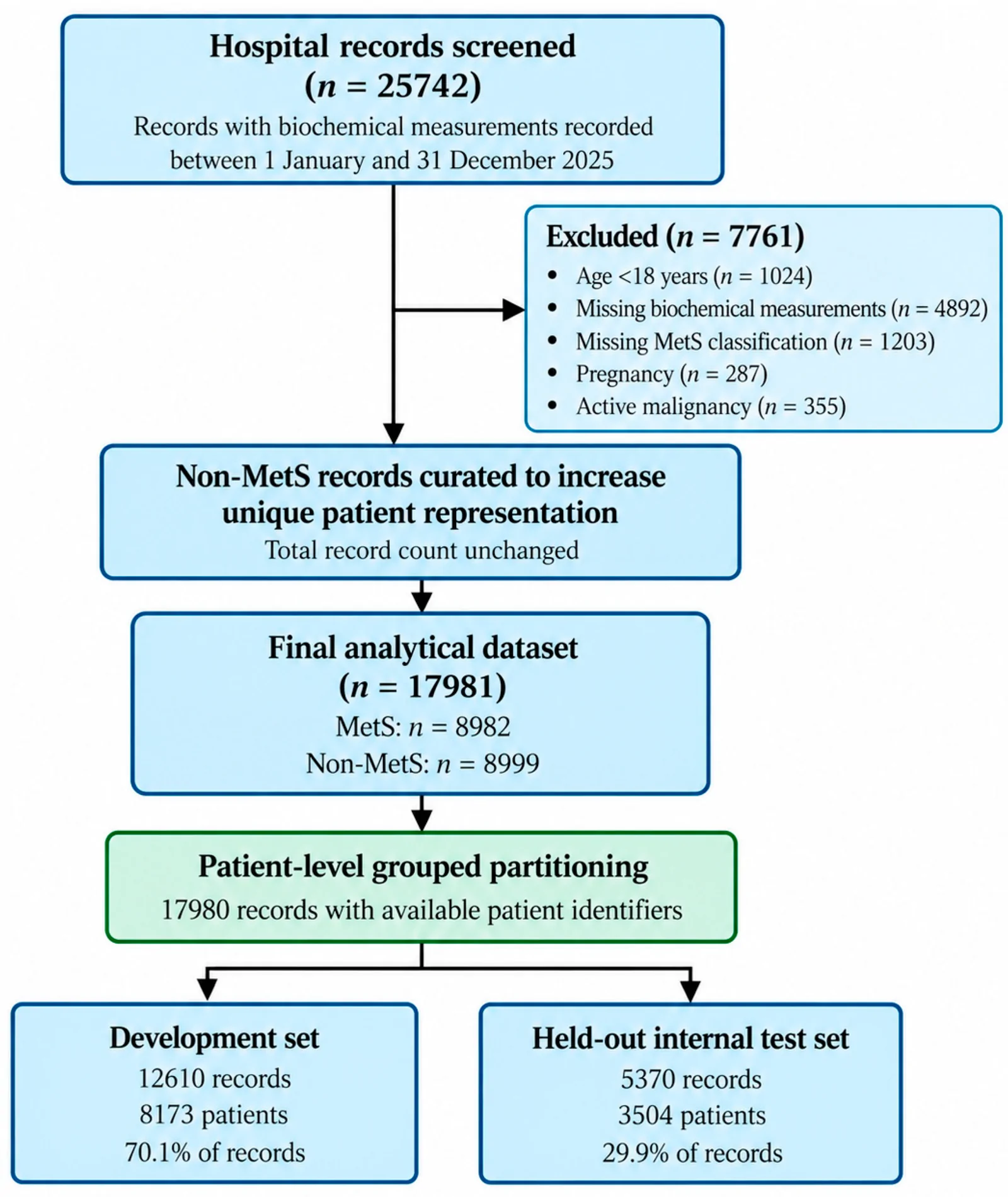
Flow chart of study population selection, analytical dataset preparation, and patient-level data partitioning. Blue boxes indicate study population screening, exclusion, data curation, final analytical dataset construction, and the resulting development and held-out internal test sets; the green box indicates patient-level grouped partitioning. MetS, metabolic syndrome. One record without an available patient identifier was not included in the patient-level partitioning procedure. Repeated records from the same patient were retained within a single partition and were not split across the development and held-out internal test sets.

This retrospective cross-sectional analysis used de-identified routine-care data from the Tokat Gaziosmanpaşa University Research and Practice Center. The source records were generated during routine clinical care between 1 January and 31 December 2025. Data extraction, de-identification, and analysis were performed after ethics approval was obtained on 5 February 2026. The final analytical dataset comprised 17,981 laboratory records, including 8982 records classified as MetS and 8999 records classified as non-MetS. Adults aged ≥18 years with complete measurements of the selected biochemical features and available MetS classification were eligible for inclusion. The exclusion criteria were pregnancy, active malignancy, and missing data for either the outcome or predictor variables. Of 25,742 hospital records screened during the study period, 7761 were excluded because of age <18 years (*n* = 1024), missing biochemical measurements (*n* = 4892), missing MetS classification (*n* = 1203), pregnancy (*n* = 287), or active malignancy *(n* = 355). The study population selection, analytical dataset preparation, and patient-level data partitioning are summarized in Figure 1.

This study was conceptualized as a laboratory-based screening analysis for reference-defined MetS rather than as a longitudinal risk-prediction study. The unit of analysis was an individual laboratory record. The dataset was split at the patient level, ensuring that all records from the same patient were assigned exclusively to either the development set or the held-out internal test set. No patient was shared between the two partitions. Repeated records could remain within a given partition, but cross-partition patient overlap was prevented.

Biochemical measurements were extracted retrospectively from routine hospital laboratory records and were analyzed using their standard clinical laboratory units. LDL cholesterol was measured directly using the routine clinical laboratory method. Detailed metadata on the assay platform and internal laboratory quality-control procedures were not included in the analytical file.

### 2.2. Ethical Considerations

Ethical approval was obtained from the Tokat Gaziosmanpaşa University Non-Interventional Clinical Research Ethics Committee (decision no. 26-MOBAEK-044; 5 February 2026). The approval covered the retrospective analysis of pre-existing de-identified laboratory records generated during routine clinical care. Data extraction, analysis, and manuscript preparation were conducted after ethics approval. The study was conducted in accordance with the Declaration of Helsinki and relevant institutional and national guidelines. The requirement for informed consent was waived because the analysis was based on retrospective, de-identified data.

### 2.3. Outcome Definition

MetS status was defined using the updated National Cholesterol Education Program Adult Treatment Panel III (NCEP ATP III) criteria based on clinical, anthropometric, and biochemical records. During data extraction from the hospital information system, only records for which all five NCEP ATP III components were available for assessment were included. Records with insufficient information to assign reference MetS status according to the prespecified NCEP ATP III criteria were excluded as having missing MetS classification. All 17,981 records included in the final analytical dataset had all five NCEP ATP III components available for reference MetS classification. The clinical, anthropometric, and biochemical components used to determine MetS status corresponded to the same clinical encounter. Records meeting at least three of the following five criteria were classified as MetS: waist circumference > 102 cm in men or >88 cm in women; triglyceride levels ≥ 150 mg/dL or treatment for elevated triglycerides; HDL cholesterol < 40 mg/dL in men or <50 mg/dL in women or treatment for reduced HDL cholesterol; blood pressure ≥ 130/85 mmHg or antihypertensive treatment; and fasting glucose ≥ 100 mg/dL or treatment for elevated glucose levels. Records meeting fewer than three of these criteria were classified as having no MetS. Waist circumference and blood pressure contributed to the reference MetS classification but were not included as predictors in the models. The primary model included HbA1c and LDL cholesterol only and therefore did not directly include the biochemical criterion components of the NCEP ATP III definition. In contrast, the criterion-component and full biochemical comparison models included glucose, triglycerides, and HDL cholesterol, which overlap with the reference MetS definition. Therefore, these comparison models were interpreted as analyses of predictor–outcome overlap rather than as independent diagnostic or etiological prediction models. Treatment-related information, when available, was incorporated into the reference MetS classification according to the NCEP ATP III criteria. When treatment information was unavailable, the relevant NCEP ATP III components were classified using the corresponding measured values alone. However, medication variables were not included as model predictors. Therefore, the machine learning models were intentionally restricted to routinely measured biochemical parameters, and the primary HbA1c–LDL cholesterol model should be interpreted as a laboratory-based screening model for reference-defined MetS rather than as a comprehensive clinical diagnostic model.

### 2.4. Biochemical Features and Model Sets

Three prespecified feature sets were evaluated. The primary model included HbA1c and LDL cholesterol, which were selected because they are routinely measured biochemical markers but are not direct components of the NCEP ATP III MetS definition. This model was used to evaluate whether HbA1c and LDL cholesterol provide screening information for reference-defined MetS without directly using the biochemical criterion components of the reference standard.

To quantify the effect of predictor–outcome overlap, two secondary comparison models were also evaluated: (i) a criterion-component model including glucose, triglycerides, and HDL cholesterol, which are biochemical components of the NCEP ATP III definition; and (ii) a full biochemical model including glucose, triglycerides, HDL cholesterol, HbA1c, and LDL cholesterol. The criterion-component and full biochemical models were interpreted as comparison models for demonstrating the influence of incorporation bias and as upper-bound performance estimates rather than as primary diagnostic or predictive models.

### 2.5. Data Preprocessing and Internal Validation

All five features were continuous and complete in the analytical dataset. The final analytical dataset included 8982 MetS records and 8999 non-MetS records. Before patient-level partitioning, the non-MetS records were curated to increase unique patient representation by reducing overrepresentation of repeated records from the same individuals, while preserving the total number of non-MetS records. This procedure did not alter the overall analytical sample size. The near-equal distribution of MetS and non-MetS records should not be interpreted as the prevalence of MetS in the source hospital population. Accordingly, the analytical dataset should be regarded as a modelling sample rather than as a consecutive sample representative of the prevalence of MetS in the source hospital population. No oversampling, undersampling, SMOTE, matching, or other artificial class-balancing procedure was applied during model development.

Before model comparison, one record without an available patient identifier was excluded from the patient-level partitioning procedure. The remaining 17,980 records were partitioned at the patient level into a development set comprising 12,610 records from 8173 patients and a held-out internal test set comprising 5370 records from 3504 patients. All records from the same patient were assigned exclusively to one partition, and no patient was shared between the development and test sets. Algorithm comparison and model selection were performed exclusively within the development set using patient-level grouped five-fold cross-validation. The held-out internal test set was kept untouched and was used only once for final performance evaluation, calibration assessment, decision curve analysis, threshold-based performance summary, and SHAP-based interpretation.

Hyperparameter tuning was performed for the evaluated machine learning algorithms within the development set using grouped five-fold cross-validation. The held-out internal test set was not used during hyperparameter tuning, algorithm selection, threshold selection, or model optimization. The optimal hyperparameter configuration for each algorithm was selected according to the mean cross-validated ROC-AUC within the development set.

Scaling was applied only to scale-sensitive algorithms and was implemented within the model-development pipelines. Accordingly, preprocessing parameters were estimated from the training data only within each cross-validation fold or, for final testing, from the development set only.

### 2.6. Model Development and Reference Models

Gradient boosting was used as the primary machine learning algorithm for the prespecified feature-set analyses. This choice was based on its ability to model nonlinear and threshold-like relationships, its strong performance in the initial development-set benchmarking, and its suitability for SHAP-based interpretation. The same gradient-boosting framework was applied to the primary HbA1c–LDL cholesterol model, the criterion-component comparison model, and the full biochemical comparison model to ensure that differences in performance reflected the predictor sets rather than changes in algorithm class. The final gradient-boosting models were implemented using the GradientBoostingClassifier in scikit-learn 1.8.0 with 200 estimators, a learning rate of 0.10, maximum tree depth of 2, and a minimum of 10 samples per leaf. The log-loss objective was used with subsample = 1.0, indicating deterministic rather than stochastic boosting, and the random state was fixed at 42. Parameters not otherwise specified retained the library defaults.

For contextual comparison, nine supervised learning algorithms were evaluated within the development set using patient-level grouped five-fold cross-validation: logistic regression, k-nearest neighbors, support vector machines, decision trees, random forests, gradient boosting, XGBoost, Gaussian naïve Bayes, and multilayer perceptrons. Preprocessing, scaling where required, hyperparameter tuning, and model fitting were implemented within algorithm-specific pipelines to avoid data leakage. Hyperparameter tuning was performed using a prespecified grid-search strategy within the development set, and the held-out internal test set was not used during preprocessing, hyperparameter tuning, algorithm selection, threshold selection, or model optimization.

Because the primary scientific focus was the evaluation of HbA1c and LDL cholesterol as screening markers and the quantification of predictor–outcome overlap, algorithm benchmarking was interpreted as a secondary methodological comparison rather than as the primary scientific result. Standard logistic regression and spline logistic regression were retained as reference models for contextual comparison with gradient boosting. The spline logistic regression model incorporated cubic spline transformations with four knots for each continuous feature and was implemented within a pipeline including spline construction, standardization, and logistic regression.

### 2.7. Performance, Calibration, and Clinical Utility

Model performance was summarized using the ROC-AUC, accuracy, precision, sensitivity, specificity, F1-score, and Brier score. Patient-level nonparametric bootstrap resampling with 1000 iterations was applied to obtain 95% confidence intervals for the AUC, paired AUC differences, threshold-based performance metrics, Brier score, calibration intercept, and calibration slope in the held-out test set. For threshold-based performance summaries, the default probability threshold of 0.50 was used for consistency across reference-model comparisons. As a screening-oriented sensitivity analysis, an additional operating threshold was derived from out-of-fold predictions obtained by patient-level grouped five-fold cross-validation within the development set to achieve approximately 90% sensitivity. This fixed threshold was then applied to the held-out internal test set without further optimization. The held-out test set was not used for model tuning, algorithm selection, or performance optimization.

As complementary specificity-oriented analyses, two operating points targeting approximately 80% and 90% specificity were derived from out-of-fold predictions obtained within the development set and then applied unchanged to the held-out internal test set. For these operating points, sensitivity and complete confusion-matrix counts were reported. Standardized partial ROC-AUC was also calculated over the high-specificity region corresponding to specificity ≥ 0.80 (false-positive rate ≤ 0.20), with 95% confidence intervals estimated using patient-level bootstrap resampling with 1000 iterations.

The calibration of the held-out test predictions was examined using a calibration plot, Brier score, and calibration intercept and slope. Calibration curves were constructed using 10 quantile-based bins of predicted probabilities. The intercept and slope were derived by regressing the observed binary outcome on the logit of the predicted probability with ideal values of 0 and 1, respectively. Decision curve analysis was performed as an exploratory assessment of potential clinical utility. In this context, a positive model classification was interpreted as a possible trigger for a complete clinical assessment for MetS, including anthropometric evaluation, blood pressure measurement, medication review, and physician assessment, rather than as an automatic diagnosis or treatment decision. Based on held-out test probabilities, the net benefits of gradient boosting, standard logistic regression, and spline logistic regression were compared with treat-all and treat-none strategies over threshold probabilities ranging from 0.05 to 0.95.

### 2.8. Explainability Methods

SHAP values were computed for the primary HbA1c–LDL cholesterol gradient-boosting model fitted to the development dataset. TreeExplainer was applied to the held-out internal test set to quantify the direction and magnitude of HbA1c and LDL cholesterol contributions on the model-output scale. Global feature importance was summarized using the mean absolute SHAP value for each feature, and a beeswarm summary plot was used to display both the magnitude and direction of contributions across individual observations. SHAP dependence plots were generated for HbA1c and LDL cholesterol to examine the shape of their contributions across the observed feature ranges. Patient-level bootstrap resampling was used to assess the stability of the global SHAP importance ranking. All SHAP summaries were based on the same set of observations. SHAP values were interpreted as explanations of the fitted model’s behavior rather than as evidence of causal effects.

### 2.9. Statistical Software and Reporting

Analyses were conducted using Python 3.13.5. Model development and evaluation were performed using scikit-learn 1.8.0 and XGBoost 3.1.3; model explanations were performed using SHAP 0.50.0; and numerical processing, data handling, statistical calculations, and visualization were performed using NumPy 2.3.5, Pandas 2.2.3, SciPy 1.17.0, and Matplotlib 3.10.8. The random state was fixed at 42, where supported. Reporting followed the TRIPOD+AI principles to ensure transparent reporting of the screening-oriented machine learning models and their internal validation (Supplementary File S1). All statistical estimates and model performance metrics were interpreted using 95% confidence intervals where applicable.

In addition, ChatGPT (OpenAI) was used only for language editing, grammar correction, formatting support, and improvement of textual clarity during manuscript preparation. No artificial intelligence tool was used for data generation, statistical analysis, model development, result interpretation, or drawing scientific conclusions.

## 3. Results

### 3.1. Dataset Characteristics

The final dataset comprised 17,981 records, including 8982 (50.0%) classified as MetS and 8999 (50.0%) classified as non-MetS. The mean age was 61.53 ± 13.61 years and did not differ materially between the MetS and non-MetS groups (61.65 ± 13.28 vs. 61.42 ± 13.94 years; *p* = 0.255; SMD = 0.017). Women accounted for 50.2% of the overall sample and were slightly more frequent in the non-MetS group than in the MetS group (52.7% vs. 47.7%; *p* < 0.001; SMD = 0.101). Records classified as MetS had higher mean glucose, HbA1c, triglyceride, and LDL cholesterol levels and lower HDL cholesterol levels than non-MetS records. The largest standardized mean differences were observed for HbA1c (SMD = 0.338), glucose (SMD = 0.312), and triglycerides (SMD = 0.309), whereas the between-group difference in LDL cholesterol was smaller (SMD = 0.123) (Table 1).

### 3.2. Primary HbA1c–LDL Cholesterol Screening Model

The primary model included HbA1c and LDL cholesterol. This model was evaluated to determine whether routinely measured biochemical markers that are not direct components of the NCEP ATP III MetS definition provide screening information for reference-defined MetS. In the patient-level held-out internal test set, the HbA1c–LDL cholesterol gradient-boosting model showed moderate-to-strong discrimination, with an ROC-AUC of 0.810, accuracy of 0.733, sensitivity of 0.824, specificity of 0.641, F1-score of 0.756, and a Brier score of 0.174 (Table 2).

These findings suggest that HbA1c and LDL cholesterol contain measurable screening information for reference-defined MetS. However, the performance was substantially lower than that of models including glucose, triglycerides, and HDL cholesterol, indicating that the strongest apparent discrimination arises when variables directly embedded in the reference criteria are included as predictors.

### 3.3. Effect of Predictor–Outcome Overlap

To quantify the influence of predictor–outcome overlap, the primary HbA1c–LDL cholesterol model was compared with two comparison models. The criterion-component comparison model included glucose, triglycerides, and HDL cholesterol, which are biochemical components of the NCEP ATP III MetS definition. In the patient-level held-out internal test set, this model achieved a higher ROC-AUC of 0.935, with an accuracy of 0.845, sensitivity of 0.789, specificity of 0.903, F1-score of 0.837, and Brier score of 0.102 (Table 2).

The full biochemical comparison model, which included glucose, triglycerides, HDL cholesterol, HbA1c, and LDL cholesterol, achieved the highest apparent performance, with an ROC-AUC of 0.956, accuracy of 0.879, sensitivity of 0.865, specificity of 0.894, F1-score of 0.878, and Brier score of 0.084 (Table 2), although this performance remained influenced by predictor–outcome overlap because three predictors were components of the reference MetS definition.

Compared with the primary HbA1c–LDL cholesterol model, the criterion-component model increased ROC-AUC by 0.125 (95% CI 0.113–0.137) and reduced the Brier score by 0.072 (95% CI 0.065–0.078). The additional improvement from the criterion-component model to the full biochemical model was smaller, with a ΔROC-AUC of 0.021 (95% CI 0.018–0.025) and a reduction in Brier score of 0.018 (95% CI 0.015–0.021). The full biochemical model exceeded the primary model by 0.146 in ROC-AUC (95% CI 0.135–0.156) and by 0.090 in Brier-score reduction (95% CI 0.084–0.095).

These comparisons indicate that most of the gain in apparent discrimination was associated with inclusion of variables directly embedded in the reference standard. The smaller increment from the criterion-component model to the full biochemical model suggests that HbA1c and LDL cholesterol added some screening information, although this comparison remains affected by incorporation bias.

### 3.4. Contextual Algorithm Benchmarking

For contextual methodological comparison, nine supervised learning algorithms were evaluated within the development set using patient-level grouped five-fold cross-validation on the full five-variable biochemical feature set. This benchmarking analysis was performed to compare algorithmic behavior under the same validation framework; however, it was not interpreted as the primary scientific result of the present study. The primary scientific focus remained the HbA1c–LDL cholesterol screening model and the comparison of feature-set performance.

In the development-set cross-validation, gradient boosting and XGBoost showed nearly identical discrimination, with mean ROC-AUC values of 0.957 ± 0.005 and 0.956 ± 0.004, respectively. Gradient boosting achieved an accuracy of 0.873 ± 0.009 and an F1-score of 0.871 ± 0.010, whereas XGBoost achieved an accuracy of 0.873 ± 0.004 and an F1-score of 0.871 ± 0.004 (Table 3). Given the minimal difference in discrimination, gradient boosting was retained as the common modelling framework for the prespecified feature-set analyses for consistency across comparisons and compatibility with the planned SHAP-based interpretation, rather than on the basis of a statistically meaningful superiority over XGBoost. Algorithm benchmarking was kept as a secondary methodological comparison.

**Table 3:** Contextual patient-level grouped five-fold cross-validated performance of nine algorithms using the full five-variable biochemical feature set.

Model	Accuracy, Mean ± SD	F1-Score, Mean ± SD	ROC-AUC, Mean ± SD
Logistic Regression	0.614 ± 0.009	0.571 ± 0.010	0.659 ± 0.009
KNN	0.796 ± 0.010	0.807 ± 0.009	0.892 ± 0.008
SVM	0.699 ± 0.006	0.715 ± 0.006	0.756 ± 0.008
Decision Tree	0.866 ± 0.004	0.864 ± 0.004	0.948 ± 0.004
Random Forest	0.865 ± 0.009	0.864 ± 0.010	0.952 ± 0.006
Gradient Boosting	0.873 ± 0.009	0.871 ± 0.010	0.957 ± 0.005
XGBoost	0.873 ± 0.004	0.871 ± 0.004	0.956 ± 0.004
Naïve Bayes	0.582 ± 0.006	0.449 ± 0.014	0.686 ± 0.010
MLP	0.840 ± 0.017	0.842 ± 0.016	0.930 ± 0.010

Note: Performance values are presented as mean ± standard deviation across patient-level grouped five-fold cross-validation folds within the development set. The benchmark was conducted using the full five-variable biochemical feature set comprising glucose, triglycerides, HDL cholesterol, HbA1c, and LDL cholesterol. The reported models used the selected hyperparameter configurations provided in Supplementary Table S1. This benchmarking analysis was performed as a contextual methodological comparison and was not interpreted as the primary scientific result of the study or as a definitive ranking of algorithm performance. Because Table 3 reports development-set cross-validation estimates, these values should not be directly compared with the held-out internal test-set estimates reported in Table 4 and Table 5. The held-out internal test set was not used during cross-validation, hyperparameter selection, or model comparison. ROC-AUC, receiver operating characteristic area under the curve; SD, standard deviation; HDL, high-density lipoprotein; LDL, low-density lipoprotein.

**Table 4:** Reference-model comparison for the primary HbA1c–LDL cholesterol model in the patient-level held-out internal test set (n = 5370).

Model	Accuracy	Sensitivity	Specificity	F1-Score	ROC-AUC (95% CI)	Brier Score
Logistic Regression	0.602	0.434	0.773	0.523	0.640 (0.624–0.655)	0.241
Spline Logistic Regression	0.665	0.632	0.699	0.655	0.686 (0.672–0.702)	0.228
Gradient Boosting	0.733	0.824	0.641	0.756	0.810 (0.799–0.821)	0.174

Note: This table presents a reference-model comparison using the primary HbA1c–LDL cholesterol feature set in the patient-level held-out internal test set. The primary model included HbA1c and LDL cholesterol only and did not directly include glucose, triglycerides, or HDL cholesterol, which are biochemical components of the NCEP ATP III MetS definition. Threshold-dependent metrics were calculated using the default probability threshold of 0.50 for cross-model comparison. This threshold was not optimized for clinical screening. The held-out internal test set was not used during model training, hyperparameter tuning, algorithm selection, or threshold selection. ROC-AUC confidence intervals were calculated using patient-level bootstrap resampling. ROC-AUC, receiver operating characteristic area under the curve; CI, confidence interval; MetS, metabolic syndrome; LDL, low-density lipoprotein.

**Table 5:** Threshold-based performance and calibration of the primary HbA1c–LDL cholesterol gradient-boosting model in the patient-level held-out internal test set.

Metric	Estimate	95% CI
ROC-AUC	0.810	0.799–0.821
Accuracy	0.733	0.720–0.746
Sensitivity	0.824	0.809–0.839
Specificity	0.641	0.622–0.659
Positive predictive value	0.698	0.674–0.722
Negative predictive value	0.783	0.761–0.803
F1-score	0.756	0.739–0.771
Brier score	0.174	0.169–0.179
Calibration intercept	0.016	−0.086–0.124
Calibration slope	1.040	0.980–1.104
True negatives	1712	–
False positives	960	–
False negatives	475	–
True positives	2223	–

Note: This table presents the threshold-based performance and calibration of the primary HbA1c–LDL cholesterol gradient-boosting model in the patient-level held-out internal test set. The primary model included HbA1c and LDL cholesterol only and did not directly include glucose, triglycerides, or HDL cholesterol, which are biochemical components of the NCEP ATP III MetS definition. Threshold-dependent metrics were calculated using the default probability threshold of 0.50. This threshold was used as a common reference for model comparison and was not optimized for clinical screening. Confidence intervals were calculated using patient-level bootstrap resampling. Calibration intercept and slope were estimated by regressing the observed outcome on the logit of the predicted probability. CI, confidence interval; ROC-AUC, receiver operating characteristic area under the curve; MetS, metabolic syndrome; LDL, low-density lipoprotein.

### 3.5. Reference-Model Comparison for the Primary Model

As a reference-model comparison, the primary HbA1c–LDL cholesterol feature set was evaluated using standard logistic regression, spline logistic regression, and gradient boosting in the patient-level held-out internal test set. This analysis was performed to determine whether the nonlinear gradient-boosting model provided higher apparent discrimination than conventional statistical reference models when only HbA1c and LDL cholesterol were used as predictors. Unlike the full biochemical comparison model, the primary HbA1c–LDL cholesterol model did not directly include glucose, triglycerides, or HDL cholesterol, which are biochemical components of the NCEP ATP III MetS definition. Therefore, this analysis was considered more appropriate for evaluating the screening signal of HbA1c and LDL cholesterol for reference-defined MetS.

**Figure 2:**
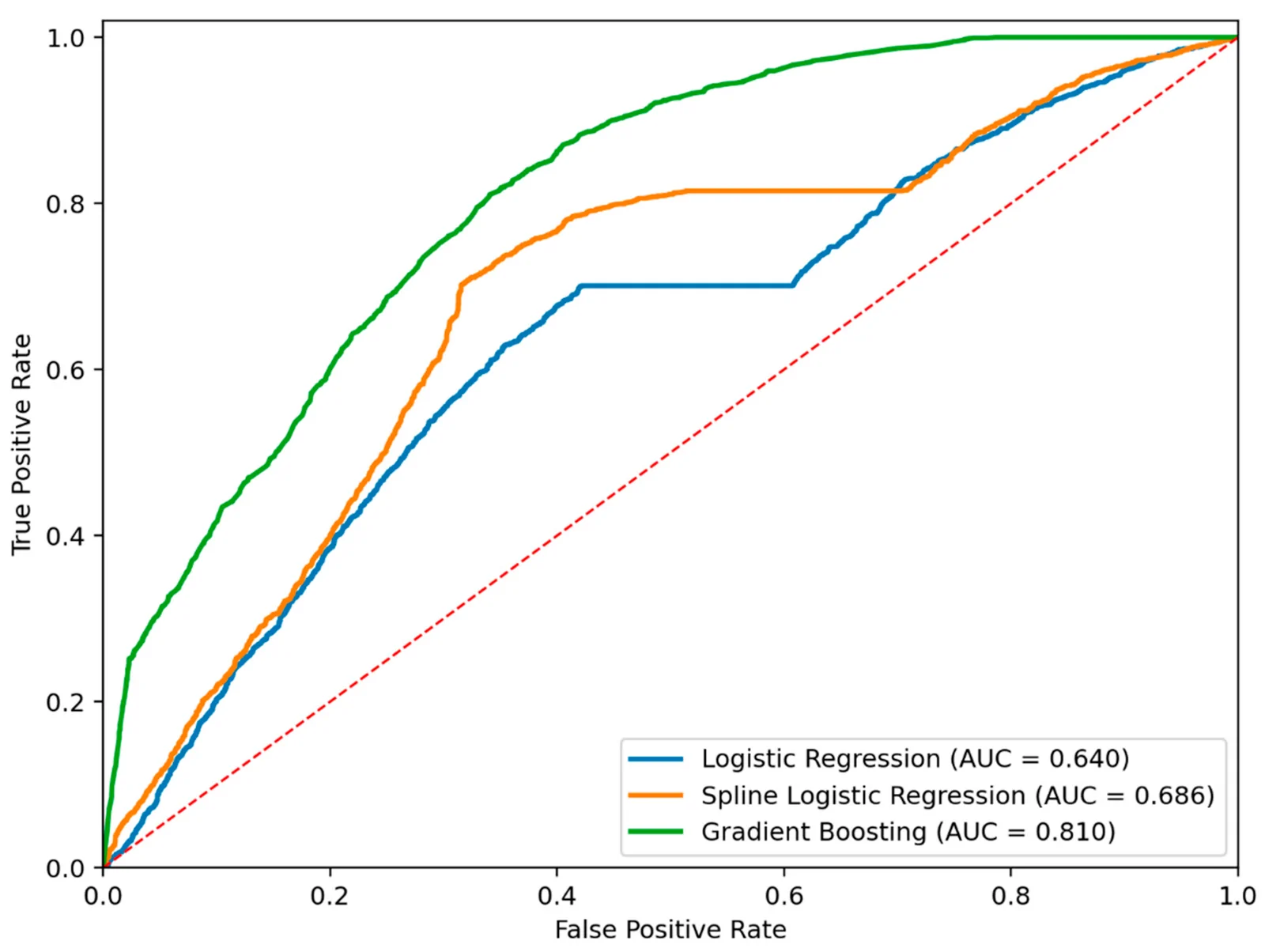
ROC curves for models using the primary HbA1c–LDL cholesterol feature set in the patient-level held-out internal test set. The primary feature set included HbA1c and LDL cholesterol only. The red dashed diagonal line represents the no-discrimination reference line (AUC = 0.50).

In the patient-level held-out internal test set, gradient boosting achieved the highest apparent performance for the primary HbA1c–LDL cholesterol feature set, with an ROC-AUC of 0.810, accuracy of 0.733, sensitivity of 0.824, specificity of 0.641, F1-score of 0.756, and Brier score of 0.174 (Table 4 and Figure 2). Standard logistic regression and spline logistic regression showed lower discrimination, with ROC-AUC values of 0.640 and 0.686, respectively (Table 4 and Figure 2). These findings suggest that nonlinear modelling captured additional screening information from HbA1c and LDL cholesterol, although the performance remained moderate and should not be interpreted as diagnostic capability.

For contextual reference, the full five-variable biochemical gradient-boosting model was also compared with standard and spline logistic regression models. Because the full biochemical feature set included glucose, triglycerides, and HDL cholesterol, which are biochemical criterion components of the NCEP ATP III MetS definition, this analysis was interpreted only as a secondary comparison rather than as independent diagnostic performance (Supplementary Table S4).

### 3.6. Calibration, Threshold-Based Performance, and Decision Curve Analysis

Calibration, threshold-based performance, confusion matrix, and decision curve analysis were evaluated for the primary HbA1c–LDL cholesterol gradient-boosting model in the patient-level held-out internal test set. Because this model included only HbA1c and LDL cholesterol and did not directly include glucose, triglycerides, or HDL cholesterol, the results were interpreted as the internal screening performance of the primary model rather than as evidence of stand-alone diagnostic capability.

Using the default probability threshold of 0.50, the primary HbA1c–LDL cholesterol gradient-boosting model correctly classified 3935 of 5370 records in the patient-level held-out internal test set. This threshold was used as a common reference for model comparison and was not optimized for clinical screening. The model achieved an ROC-AUC of 0.810, accuracy of 0.733, sensitivity of 0.824, specificity of 0.641, positive predictive value of 0.698, negative predictive value of 0.783, F1-score of 0.756, and Brier score of 0.174 (Table 5). The confusion matrix yielded 1712 true negatives, 2223 true positives, 960 false positives, and 475 false negatives.

In the screening-oriented sensitivity analysis, the development-derived probability threshold of 0.400 yielded a sensitivity of 0.901 and specificity of 0.548 in the held-out internal test set, with a positive predictive value of 0.668 and a negative predictive value of 0.846. At this threshold, 267 false-negative and 1208 false-positive classifications were observed.

In complementary specificity-oriented analyses, the development-derived operating point targeting approximately 80% specificity corresponded to a probability threshold of 0.634. When applied unchanged to the held-out internal test set, sensitivity was 0.592 (95% CI 0.572–0.611) and specificity was 0.804 (95% CI 0.789–0.818), with 2147 true negatives, 525 false positives, 1102 false negatives, and 1596 true positives. At the operating point targeting approximately 90% specificity, the corresponding threshold was 0.666; sensitivity was 0.398 (95% CI 0.380–0.418) and specificity was 0.908 (95% CI 0.898–0.918), with 2426 true negatives, 246 false positives, 1623 false negatives, and 1075 true positives. Within the high-specificity region corresponding to specificity ≥ 0.80 (false-positive rate ≤ 0.20), the standardized partial ROC-AUC was 0.664 (95% CI 0.651–0.677) (Supplementary Table S5).

**Figure 3:**
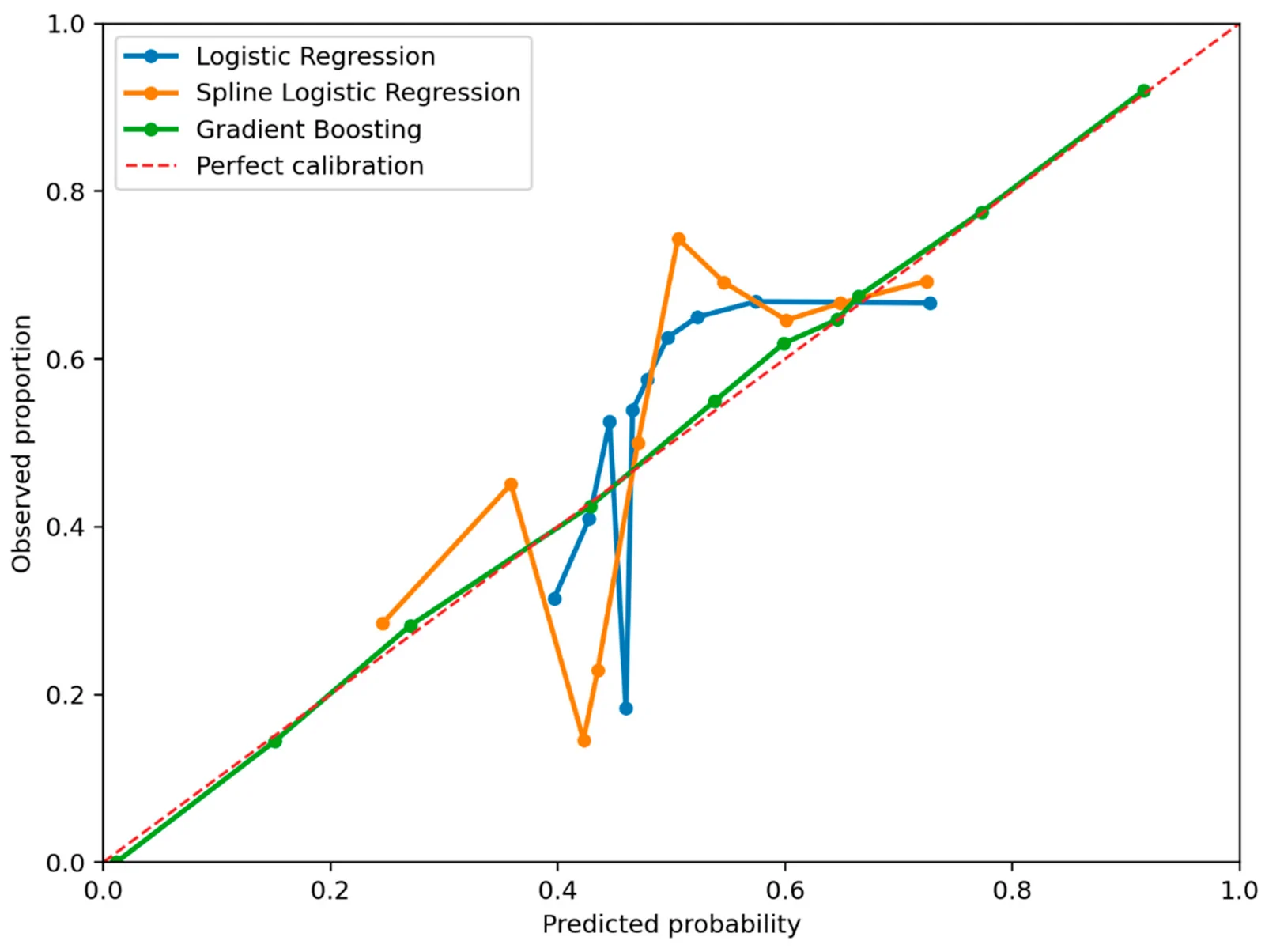
Calibration plots for the primary HbA1c–LDL cholesterol models in the patient-level held-out internal test set. The primary feature set included HbA1c and LDL cholesterol only. Calibration curves are shown for gradient boosting, standard logistic regression, and spline logistic regression.

Calibration analysis showed a calibration intercept of 0.016 and a calibration slope of 1.040 for the gradient-boosting model (Table 5). In the reference-model comparison, standard logistic regression and spline logistic regression showed Brier scores of 0.241 and 0.228, respectively, whereas gradient boosting showed a lower Brier score of 0.174. The calibration plots of the three models are shown in Figure 3.

**Figure 4:**
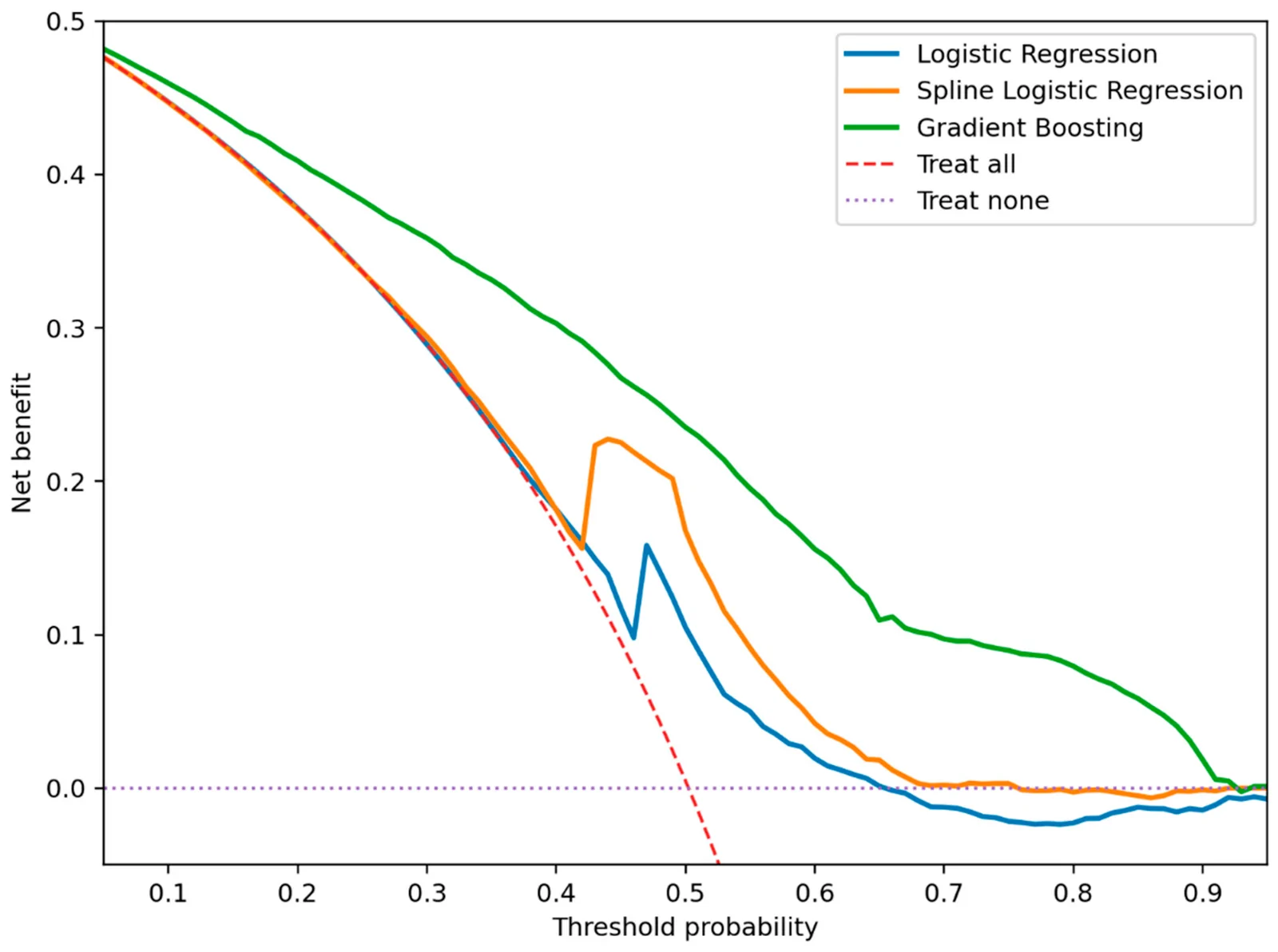
Decision curve analysis for the primary HbA1c–LDL cholesterol models in the patient-level held-out internal test set. Net benefit was compared across gradient boosting, standard logistic regression, spline logistic regression, treat-all, and treat-none strategies.

Exploratory decision curve analysis suggested that the gradient-boosting model provided higher net benefit than standard logistic regression, spline logistic regression, treat-all, and treat-none strategies across several threshold probabilities. However, this finding should be interpreted as internal exploratory evidence only and not as support for immediate clinical implementation (Figure 4).

### 3.7. Explainability Results

SHAP analysis was performed for the primary HbA1c–LDL cholesterol gradient-boosting model in the patient-level held-out internal test set. Because this model included only HbA1c and LDL cholesterol, SHAP values were interpreted as explanations of the model’s screening behavior rather than as evidence of causal or diagnostic importance. For the gradient-boosting model, SHAP values were expressed on the raw model-output (log-odds) scale.

**Figure 5:**
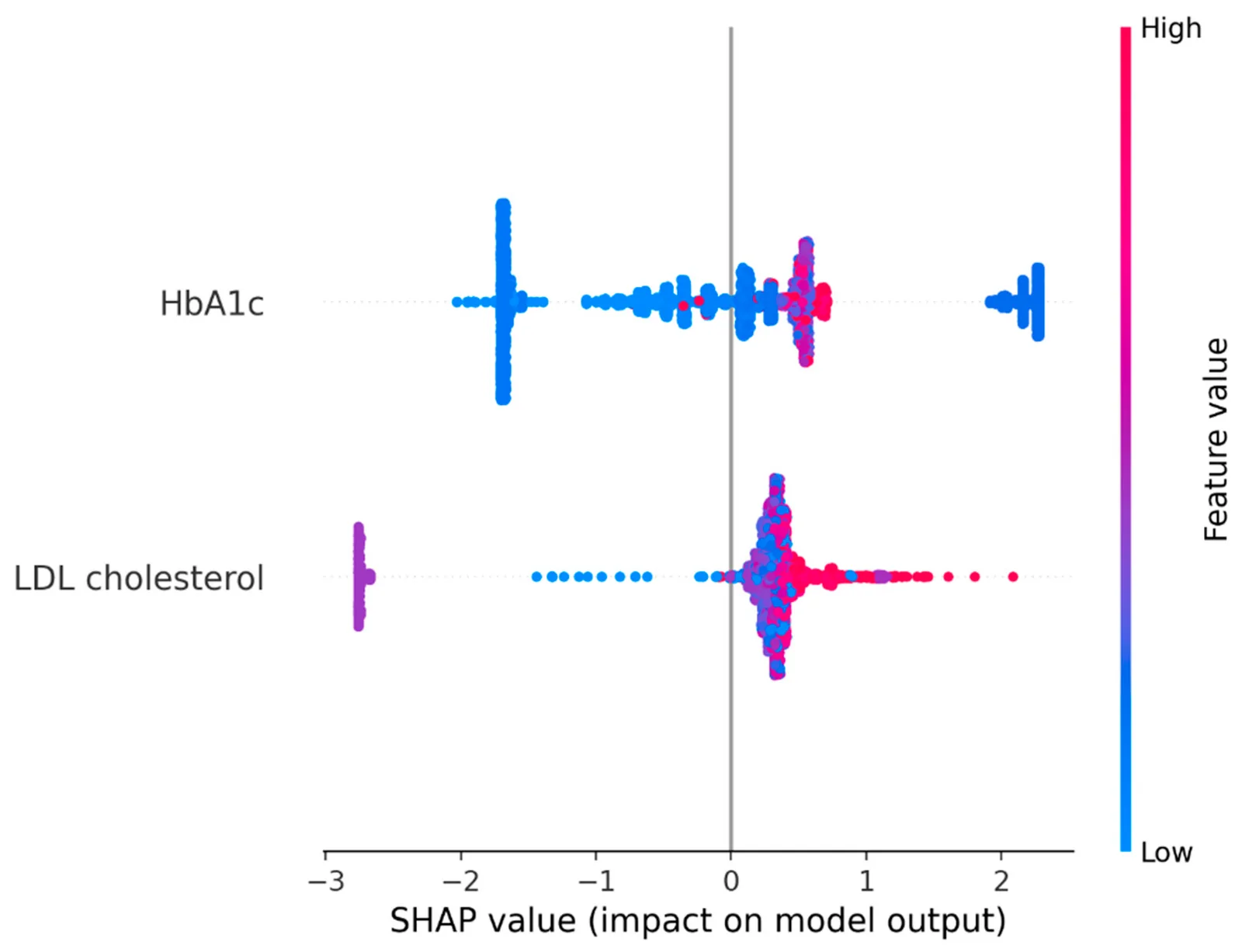
SHAP summary plot for the primary HbA1c–LDL cholesterol gradient-boosting model in the patient-level held-out internal test set. The primary feature set included HbA1c and LDL cholesterol only. SHAP values are expressed on the raw model-output (log-odds) scale and indicate the direction and magnitude of each feature’s contribution to the model output.

HbA1c showed a stronger contribution to model predictions than LDL cholesterol, with mean absolute SHAP values of 0.934 and 0.639, respectively. Higher HbA1c values generally shifted model outputs toward the reference-defined MetS class, whereas LDL cholesterol had a smaller but measurable contribution. The SHAP summary plot is shown in Figure 5. SHAP dependence plots for HbA1c and LDL cholesterol are provided in Supplementary Figures S1 and S2, respectively. Patient-level bootstrap analysis showed that this ranking was stable, with HbA1c having a higher mean absolute SHAP value than LDL cholesterol in all bootstrap samples.

These findings suggest that the screening signal of the primary model was mainly associated with long-term glycemic exposure, with additional but more limited information from LDL cholesterol.

## 4. Discussion

This study evaluated whether HbA1c and LDL cholesterol provide screening information for reference-defined MetS and examined how model performance changes when biochemical components of the MetS definition are included as predictors. The primary HbA1c–LDL cholesterol model showed moderate-to-strong internal discrimination in the patient-level held-out test set (ROC-AUC 0.810), whereas models including glucose, triglycerides, and HDL cholesterol achieved substantially higher apparent performance. Because these latter variables are direct components of the NCEP ATP III reference definition, their higher performance was interpreted as evidence of predictor–outcome overlap rather than as independent diagnostic capability. Accordingly, the main contribution of this study is not to propose a biochemical-only diagnostic model for MetS, but to provide an explainable and internally validated screening-oriented framework that explicitly demonstrates the influence of incorporation bias.

In the reference-model comparison for the primary HbA1c–LDL cholesterol feature set, gradient boosting achieved higher apparent discrimination than standard logistic regression and spline logistic regression in the patient-level held-out internal test set (ROC-AUC 0.810 vs. 0.640 and 0.686, respectively). This suggests that nonlinear modelling may capture additional screening information from HbA1c and LDL cholesterol beyond conventional linear or spline-based statistical models. Tree-based boosting methods are well-suited to modelling nonlinear and threshold-like patterns that may be difficult to pre-specify in conventional regression models [17,18]. This modelling capacity is relevant to MetS because the syndrome reflects the interaction of multiple metabolic abnormalities rather than a single isolated defect [29]. However, the performance of the primary model remained moderate and should not be interpreted as diagnostic capability. The substantially higher AUC observed in the full five-variable biochemical comparison model (ROC-AUC 0.956) should be considered an upper-bound estimate influenced by predictor–outcome overlap, because glucose, triglycerides, and HDL cholesterol were also components of the reference MetS definition. Therefore, the role of gradient boosting in this study is best understood as an internally evaluated modelling framework for screening signal detection rather than as evidence of a clinically ready diagnostic tool.

The threshold-based performance of the primary HbA1c–LDL cholesterol model should be interpreted in light of the distinction between discrimination and classification at a selected cutoff. ROC-AUC is a threshold-independent measure of the model’s ability to rank MetS and non-MetS records, whereas sensitivity, specificity, positive predictive value, and negative predictive value depend on the selected probability threshold [19,20]. In the present analysis, threshold-dependent metrics were calculated using the default probability threshold of 0.50 for consistency across the reference-model comparisons. This threshold was not optimized for clinical screening and should be interpreted only as a common reference operating point. At the default 0.50 threshold, the primary gradient-boosting model showed higher sensitivity than specificity (0.824 vs. 0.641), with 960 false-positive and 475 false-negative classifications. A sensitivity-oriented analysis using a development-derived probability threshold of 0.400 increased sensitivity to 0.901, while specificity decreased to 0.548. This reduced the number of false-negative classifications from 475 to 267, at the cost of increasing false-positive classifications from 960 to 1208. The 0.400 threshold should therefore be interpreted as a development-derived, screening-oriented operating point rather than as a clinically validated cutoff. The resulting 1208 false-positive classifications underscore the additional downstream assessment that a sensitivity-prioritized screening strategy could generate. Conversely, the higher-specificity operating points reduced false-positive classifications to 525 and 246, respectively, but at the cost of lower sensitivities of 0.592 and 0.398, illustrating the trade-off between case detection and downstream referral burden. These classification errors would have different practical implications in a screening context: false-positive results could increase the number of individuals referred for complete clinical assessment, whereas false-negative results could leave some individuals without further evaluation. Accordingly, model-positive classifications should prompt further clinical assessment rather than direct diagnostic action.

The feature-set comparison was central to addressing the main conceptual limitation of the study. Glucose, triglycerides, and HDL cholesterol are biochemical components of the updated NCEP ATP III definition; therefore, models including these variables partly reconstruct the reference standard [7]. The criterion-component comparison model achieved an AUC of 0.935, confirming that much of the apparent discriminative performance arose from variables directly embedded in the outcome definition. Compared with the primary HbA1c–LDL cholesterol model, the criterion-component model increased ROC-AUC by 0.125 (95% CI 0.113–0.137) and reduced the Brier score by 0.072 (95% CI 0.065–0.078). The full five-variable biochemical comparison model increased the AUC to 0.956, with a further ΔROC-AUC of 0.021 (95% CI 0.018–0.025) and a Brier-score reduction of 0.018 (95% CI 0.015–0.021) relative to the criterion-component model. This smaller incremental improvement suggests that HbA1c and LDL cholesterol may contribute some additional screening information, although this comparison remains influenced by incorporation bias because three predictors in the full model were components of the reference standard. Consequently, the findings are more appropriately interpreted as evidence of screening signal and incorporation-bias effects rather than as early risk prediction, causal risk assessment, or stand-alone MetS diagnosis.

For the primary HbA1c–LDL cholesterol model, calibration analysis showed an intercept close to zero and a slope slightly greater than one, suggesting acceptable internal calibration, although recalibration may be required in external populations [30]. The calibration intercept was 0.016, and the calibration slope was 1.040 in the patient-level held-out internal test set. The Brier score was lower for gradient boosting than for standard and spline logistic regression (0.174 vs. 0.241 and 0.228, respectively), indicating better overall probability estimation within the held-out internal test set. Exploratory decision curve analysis also suggested that the gradient-boosting model provided higher net benefit than the reference models across a range of threshold probabilities [31]. However, these findings should be interpreted cautiously because they were derived from an internal, single-center, patient-level held-out test set and a near-balanced analytical sample. The decision-curve findings should therefore be considered exploratory rather than evidence of established clinical utility. In this context, a positive model classification should be viewed only as a possible trigger for complete clinical assessment of MetS, including anthropometric evaluation, blood pressure measurement, medication review, and physician assessment, rather than as an automatic diagnosis or treatment decision.

Although HbA1c is not a direct component of the NCEP ATP III MetS definition, it is biologically and clinically related to fasting glucose, which is one of the reference criteria. Therefore, the primary HbA1c–LDL cholesterol model cannot be considered fully independent of the reference standard. Part of its observed discrimination may reflect this indirect association between HbA1c and the glucose criterion, introducing a residual form of incorporation bias. The primary model’s performance should therefore be interpreted as internal screening discrimination rather than as evidence of completely independent diagnostic information.

SHAP analysis enhanced the transparency of the primary HbA1c–LDL cholesterol gradient-boosting model by showing how each predictor contributed to model outputs. HbA1c showed a stronger contribution than LDL cholesterol, with mean absolute SHAP values of 0.934 and 0.639, respectively, in the patient-level held-out internal test set, suggesting that the model’s screening signal was mainly associated with long-term glycemic exposure, with a smaller contribution from LDL cholesterol. SHAP values were expressed on the raw model-output (log-odds) scale. Dependence plots were additionally examined to characterize how SHAP contributions varied across the observed ranges of HbA1c and LDL cholesterol; any apparent changes in these plots should not be interpreted as clinically validated biomarker thresholds. This finding is consistent with the biological relevance of glycemic dysregulation in MetS, but it should not be interpreted as evidence of causal or diagnostic importance. SHAP is a model-explanation method that quantifies how individual predictors contribute to a fitted model’s output [25]. Practical guidance on SHAP interpretation emphasizes that these explanations should be understood as summaries of model behavior rather than evidence of causal or etiological importance [27]. Similar interpretability approaches have also been applied in MetS-related machine learning research [28].

The study has several strengths, including a large analytical sample, a clearly defined patient-level development and held-out internal test framework, comparison of multiple algorithms, reference-model comparisons using standard and spline logistic regression, bootstrap confidence intervals, calibration assessment, exploratory decision curve analysis, prespecified feature-set comparisons, and SHAP-based model interpretation. These analyses improved methodological transparency and helped distinguish the screening signal provided by HbA1c and LDL cholesterol from the performance inflation observed when diagnostic criterion components were included as predictors.

This study has several important limitations. First, although the primary HbA1c–LDL cholesterol model did not directly include glucose, triglycerides, or HDL cholesterol, the reference MetS outcome was still defined according to clinical and biochemical criteria derived from routine records. Moreover, HbA1c is closely related to glycemic status and therefore indirectly related to fasting glucose, which is itself a component of the reference MetS definition. Thus, some residual incorporation bias may remain even in the primary model. Therefore, the primary model should be interpreted as a screening model for reference-defined MetS rather than as an independent diagnostic or etiological prediction model. In addition, the criterion-component and full biochemical comparison models were affected by incorporation bias because glucose, triglycerides, and HDL cholesterol were both predictors and components of the NCEP ATP III reference definition. Accordingly, the higher discriminatory performance of these comparison models should be interpreted as evidence of predictor–outcome overlap and not as independent diagnostic capability. Second, external, temporal, and multicenter validation was not performed; therefore, model performance may vary across populations, laboratory platforms, diagnostic practices, and case-mix distributions. The present findings should therefore be considered hypothesis-generating and restricted to internal validation within a single-center retrospective dataset. No implication of clinical readiness, broad applicability, or immediate implementation should be inferred from the current results. Third, several clinically relevant variables, including age, sex, waist circumference, blood pressure, medication use, smoking status, and comorbidities, were not included as model inputs. Although waist circumference and blood pressure contributed to the reference MetS classification, their raw values were not retained as analyzable variables for independent recategorization. Therefore, we could not repeat the analysis using a newly derived MetS outcome based explicitly on waist circumference and blood pressure, as would be desirable for a stronger clinically anchored validation of a biochemical screening model. Fourth, although development and test sets were separated at the patient level and no patient was shared between the two partitions, repeated records from the same patient could remain within a given partition. Such within-patient repetition may reduce the effective amount of independent information and may influence estimated model precision. This within-patient dependence may also represent a departure from the conventional independent and identically distributed assumption at the record level. Patient-level grouped cross-validation and patient-level bootstrap resampling were used to reduce this concern, but future studies using one observation per patient or longitudinal modelling would provide a stricter assessment of generalizability. Fifth, detailed laboratory metadata, including assay platforms, instruments, and internal quality-control procedures, were unavailable. Although LDL cholesterol was measured directly, detailed information on the specific assay platform and analytical performance characteristics was not available in the analytical dataset. This limitation is particularly relevant in dyslipidemic and triglyceride-rich states, because direct homogeneous LDL cholesterol assays may show method-dependent bias when lipoprotein composition is altered. Different direct LDL cholesterol assays have also been reported to show variable specificity and nonselectivity in the presence of abnormal lipoproteins or elevated triglyceride concentrations [32,33]. Sixth, although hyperparameter tuning was performed within the development set, the search spaces were intentionally limited and not equally broad across all algorithms. Therefore, the algorithm comparison should be interpreted as a contextual comparison within prespecified search spaces rather than as a definitive ranking of all possible optimized model configurations. Seventh, decision curve analysis was based on internal test-set predictions and should be interpreted as exploratory rather than evidence of clinical effectiveness. In addition, the near-equal distribution of MetS and non-MetS records in the analytical dataset does not represent the prevalence of MetS in the source population. Consequently, prevalence-dependent measures such as positive and negative predictive values, as well as calibration and decision-curve findings, may differ in populations with different MetS prevalence. Eighth, formal runtime, memory use, and deployment-related complexity benchmarking were not performed. Threshold-dependent performance was summarized at the default 0.50 probability threshold and supplemented by development-derived sensitivity- and specificity-oriented operating points. These thresholds were intended for internal screening-oriented evaluation and should not be interpreted as clinically validated cutoffs. Finally, the retrospective design precludes conclusions about prospective clinical impact; therefore, recalibration, external validation, and implementation studies are required.

## 5. Conclusions

The primary HbA1c–LDL cholesterol gradient-boosting model provided moderate-to-strong internal screening performance for reference-defined MetS in a single-center retrospective dataset. Because this model did not directly include glucose, triglycerides, or HDL cholesterol, it reduced the direct predictor–outcome overlap present in models using biochemical criterion components. However, because HbA1c is closely related to glycemic status and indirectly related to the fasting glucose component of the reference definition, some residual incorporation bias cannot be excluded. Its performance should be interpreted as a screening signal rather than as evidence of stand-alone diagnostic capability.

The substantially higher performance observed in the criterion-component and full biochemical comparison models was consistent with a substantial influence of incorporation bias when variables that are part of the NCEP ATP III definition are used as predictors. Therefore, the high apparent discrimination of models including glucose, triglycerides, and HDL cholesterol should not be interpreted as independent diagnostic or predictive performance.

Overall, this study should be viewed as an explainable, internally validated, screening-oriented machine learning analysis of HbA1c and LDL cholesterol rather than as a clinically ready diagnostic model for MetS. The findings are hypothesis-generating and limited to internal validation in a single-center, patient-level partitioned retrospective dataset. External, temporal, multicenter, and prospective validation studies are required before any clinical implementation can be considered. Future studies should use datasets that include waist circumference and blood pressure measurements as analyzable variables, allowing independent clinical recategorization of MetS before evaluating biochemical screening models.

## Figures and Tables

**Table 1:** Participant and biochemical characteristics according to metabolic syndrome status.

Variable	Overall (*n* = 17,981)	No MetS (*n* = 8999)	MetS (*n* = 8982)	*p*-Value	SMD
Age (years), mean ± SD (min–max)	61.53 ± 13.61 (18–101)	61.42 ± 13.94 (18–99)	61.65 ± 13.28 (18–101)	0.255	0.017
Sex, *n* (%)				<0.001	0.101
Female, *n* (%)	9020 (50.2)	4740 (52.7)	4280 (47.7)		
Male, *n* (%)	8961 (49.8)	4259 (47.3)	4702 (52.3)		
Glucose (mg/dL), mean ± SD	125.88 ± 58.52	116.85 ± 50.06	134.92 ± 64.66	<0.001	0.312
HbA1c (%), mean ± SD	6.46 ± 1.47	6.22 ± 1.35	6.71 ± 1.54	<0.001	0.338
Triglycerides (mg/dL), mean ± SD	159.94 ± 114.30	142.48 ± 85.69	177.44 ± 134.86	<0.001	0.309
HDL cholesterol (mg/dL), mean ± SD	46.83 ± 9.72	48.06 ± 10.87	45.60 ± 8.22	<0.001	−0.255
LDL cholesterol (mg/dL), mean ± SD	116.33 ± 39.28	113.93 ± 34.93	118.74 ± 43.08	<0.001	0.123

Note: Continuous variables are presented as mean ± standard deviation and were compared between groups using the independent-samples *t*-test. Categorical variables are presented as n (%) and were compared using the chi-square test. Standardized mean differences were calculated to quantify the magnitude of between-group differences. SD, standard deviation; SMD, standardized mean difference; MetS, metabolic syndrome; HDL, high-density lipoprotein; LDL, low-density lipoprotein.

**Table 2:** Performance of the primary and comparison gradient-boosting feature-set models in the held-out internal test set.

Panel A. Model Performance						
Feature Set	Accuracy (95% CI)	Sensitivity (95% CI)	Specificity (95% CI)	F1-Score	ROC-AUC	Brier Score
Primary HbA1c–LDL cholesterol model	0.733 (0.720–0.746)	0.824 (0.809–0.839)	0.641 (0.622–0.659)	0.756	0.810	0.174
Criterion-component comparison model	0.845 (0.835–0.855)	0.789 (0.772–0.804)	0.903 (0.891–0.915)	0.837	0.935	0.102
Full biochemical comparison model	0.879 (0.870–0.888)	0.865 (0.850–0.877)	0.894 (0.882–0.906)	0.878	0.956	0.084
**Panel B. Pairwise Differences in Discrimination and Prediction Error**						
**Comparison**			**ΔAUC**	**95% CI**	**ΔBrier**	**95% CI**
Criterion-component vs. Primary			0.125	0.113–0.137	−0.072	−0.078 to −0.065
Full biochemical vs. Criterion-component			0.021	0.018–0.025	−0.018	−0.021 to −0.015
Full biochemical vs. Primary			0.146	0.135–0.156	−0.090	−0.095 to −0.084

Note: The primary model included HbA1c and LDL cholesterol. The criterion-component comparison model included glucose, triglycerides, and HDL cholesterol, which are biochemical components of the NCEP ATP III MetS definition. The full biochemical comparison model included all five variables: glucose, triglycerides, HDL cholesterol, HbA1c, and LDL cholesterol. The criterion-component and full biochemical models were interpreted as comparison models for demonstrating the influence of predictor–outcome overlap and as upper-bound performance estimates rather than as primary diagnostic or predictive models. Performance estimates were obtained from the patient-level held-out internal test set. Confidence intervals for accuracy, sensitivity, and specificity were estimated using patient-level bootstrap resampling with 1000 iterations. Pairwise differences in ROC-AUC and Brier score were estimated using patient-level bootstrap resampling with 1000 iterations. ROC-AUC, receiver operating characteristic area under the curve; CI, confidence interval; MetS, metabolic syndrome; HDL, high-density lipoprotein; LDL, low-density lipoprotein.

## Data Availability

The data supporting the findings of this study are available from the corresponding author upon reasonable request, subject to institutional and ethical restrictions.

## Supplementary Materials

The following supporting information can be downloaded at: https://www.mdpi.com/article/10.3390/diagnostics16183048/s1, Supplementary File S1: Completed TRIPOD+AI checklist; Supplementary Table S1: Algorithm-specific preprocessing, hyperparameter search spaces, and selected hyperparameters used for contextual model benchmarking; Supplementary Table S2: Leave-one-feature-out analysis for the full five-variable biochemical comparison model; Supplementary Table S3: Logistic regression coefficients for the primary HbA1c–LDL cholesterol feature set; Supplementary Table S4: Contextual reference-model comparison for the full five-variable biochemical model in the held-out internal test set. Supplementary Table S5: Screening-oriented threshold sensitivity analysis for the primary HbA1c–LDL cholesterol gradient-boosting model. Supplementary Figure S1: SHAP dependence plot for HbA1c in the patient-level held-out internal test set. Supplementary Figure S2: SHAP dependence plot for LDL cholesterol in the patient-level held-out internal test set.

## References

[1] Hossain M.F., Hossain S., Akter M.N., Nahar A., Liu B., Faruque M.O. (2024). Metabolic syndrome predictive modelling in Bangladesh applying machine learning approach. PLoS ONE.

[2] Jiamsripong P., Mookadam M., Honda T., Khandheria B.K., Mookadam F. (2008). The metabolic syndrome and cardiovascular disease: Part I. Prev. Cardiol..

[3] Li C., Ford E.S. (2006). Definition of the metabolic syndrome: What’s new and what predicts risk?. Metab. Syndr. Relat. Disord..

[4] Grundy S.M., Benjamin I.J., Burke G.L., Chait A., Eckel R.H., Howard B.V., Mitch W., Smith S.C., Sowers J.R. (1999). Diabetes and cardiovascular disease: A statement for healthcare professionals from the American Heart Association. Circulation.

[5] Gesteiro E., Megía A., Guadalupe-Grau A., Fernandez-Veledo S., Vendrell J., González-Gross M. (2021). Early identification of metabolic syndrome risk: A review of reviews and proposal for defining pre-metabolic syndrome status. Nutr. Metab. Cardiovasc. Dis..

[6] Reasner C. (2016). The metabolic syndrome: Identification and management of the patient at high risk for cardiovascular disease. Diabetes and Cardiovascular Disease.

[7] Alberti K.G.M.M., Eckel R.H., Grundy S.M., Zimmet P.Z., Cleeman J.I., Donato K.A., Fruchart J.-C., James W.P.T., Loria C.M., Smith S.C. (2009). Harmonizing the metabolic syndrome: A joint interim statement of the International Diabetes Federation Task Force on Epidemiology and Prevention; National Heart, Lung, and Blood Institute; American Heart Association; World Heart Federation; International Atherosclerosis Society; and International Association for the Study of Obesity. Circulation.

[8] Simmons R.K., Alberti K.G.M.M., Gale E.A.M., Colagiuri S., Tuomilehto J., Qiao Q., Ramachandran A., Tajima N., Brajkovich Mirchov I., Ben-Nakhi A. (2010). The metabolic syndrome: Useful concept or clinical tool? Report of a WHO Expert Consultation. Diabetologia.

[9] DeBoer M.D. (2017). Clinical utility of metabolic syndrome severity scores: Considerations for practitioners. Diabetes Metab. Syndr. Obes..

[10] Shin H., Shim S., Oh S. (2023). Machine learning-based predictive model for prevention of metabolic syndrome. PLoS ONE.

[11] Sung K.C., Rhee E.J. (2007). Glycated haemoglobin as a predictor for metabolic syndrome in non-diabetic Korean adults. Diabet. Med..

[12] Robberecht H., Hermans N. (2016). Biomarkers of metabolic syndrome: Biochemical background and clinical significance. Metab. Syndr. Relat. Disord..

[13] Lai H., Huang H., Keshavjee K., Guergachi A., Gao X. (2019). Predictive models for diabetes mellitus using machine learning techniques. BMC Endocr. Disord..

[14] Nikityuk D.V., Klochkova S.V., Alexeeva N.T., Karpova A.V. (2024). Anthropometric indices in predicting the risks of occurrence and outcomes of diseases at present stage. J. Anat. Histopathol..

[15] Kavakiotis I., Tsave O., Salifoglou A., Maglaveras N., Vlahavas I., Chouvarda I. (2017). Machine learning and data mining methods in diabetes research. Comput. Struct. Biotechnol. J..

[16] Boehm B.O., Claudi-Boehm S. (2005). The metabolic syndrome. Scand. J. Clin. Lab. Investig..

[17] Natekin A., Knoll A. (2013). Gradient boosting machines, a tutorial. Front. Neurorobotics.

[18] Chen T., Guestrin C. XGBoost: A scalable tree boosting system. Proceedings of the 22nd ACM SIGKDD International Conference on Knowledge Discovery and Data Mining.

[19] Carrington A.M., Manuel D.G., Fieguth P.W., Ramsay T., Osmani V., Wernly B., Bennett C., Hawken S., McInnes M., Magwood O. (2023). Deep ROC analysis and AUC as balanced average accuracy for improved classifier selection, audit and explanation. IEEE Trans. Pattern Anal. Mach. Intell..

[20] Huang J., Ling C.X. (2005). Using AUC and accuracy in evaluating learning algorithms. IEEE Trans. Knowl. Data Eng..

[21] Cawley G.C., Talbot N.L.C. (2010). On over-fitting in model selection and subsequent selection bias in performance evaluation. J. Mach. Learn. Res..

[22] Powers D.M.W., Atyabi A. The problem of cross-validation: Averaging and bias, repetition and significance. Proceedings of the 2012 Spring Congress on Engineering and Technology.

[23] Arjunan G. (2021). Implementing explainable AI in healthcare: Techniques for interpretable machine learning models in clinical decision-making. Int. J. Sci. Res. Manag..

[24] Schwartz J., Cato K. Machine learning based clinical decision support and clinician trust. Proceedings of the 2020 IEEE International Conference on Healthcare Informatics.

[25] Lundberg S.M., Lee S.-I. (2017). A unified approach to interpreting model predictions. Adv. Neural Inf. Process. Syst..

[26] Wali K. (2020). A comprehensive survey on explainable artificial intelligence: Methods, challenges, and future directions. Int. J. Sci. Technol..

[27] Ponce-Bobadilla A.V., Schmitt V., Maier C.S., Mensing S., Stodtmann S. (2024). Practical guide to SHAP analysis: Explaining supervised machine learning model predictions in drug development. Clin. Transl. Sci..

[28] Zhang Y., Zhang X., Razbek J., Li D., Xia W., Bao L., Mao H., Daken M., Cao M. (2022). Opening the black box: Interpretable machine learning for predictor finding of metabolic syndrome. BMC Endocr. Disord..

[29] Anderson J.J.B., Prytherch S.A., Sparling M., Barrett C., Guyton J.R. (2006). The metabolic syndrome. Nutr. Today.

[30] Van Calster B., McLernon D.J., van Smeden M., Wynants L., Steyerberg E.W., Topic Group ‘Evaluating diagnostic tests and prediction models’ of the STRATOS initiative (2019). Calibration: The Achilles heel of predictive analytics. BMC Med..

[31] Vickers A.J., Elkin E.B. (2006). Decision curve analysis: A novel method for evaluating prediction models. Med. Decis. Mak..

[32] Wolska A., Remaley A.T. (2020). Measuring LDL-cholesterol: What is the best way to do it?. Curr. Opin. Cardiol..

[33] Langlois M.R., Chapman M.J., Cobbaert C., Mora S., Remaley A.T., Ros E., Watts G.F., Borén J., Baum H., Bruckert E. (2018). Quantifying Atherogenic Lipoproteins: Current and Future Challenges in the Era of Personalized Medicine and Very Low Concentrations of LDL Cholesterol. A Consensus Statement from EAS and EFLM. Clin. Chem..

