# Supplementary material for: Screening Signals of Reference-Defined Metabolic Syndrome Using HbA1c and LDL Cholesterol: An Explainable Machine Learning Study
Source: Diagnostics (Basel). 2026 Sep 20;16(18):3048. doi: 10.3390/diagnostics16183048 (PMC13605401; doi:10.3390/diagnostics16183048)

Supplementary Materials

The completed TRIPOD+AI checklist is provided as Supplementary File S1. Algorithm-specific preprocessing, hyperparameter search spaces, and selected hyperparameters used for contextual model benchmarking are provided in Supplementary Table S1. Leave-one-feature-out analyses for the full five-variable biochemical comparison model are provided in Supplementary Table S2. Patient-clustered logistic regression coefficients for the primary HbA1c–LDL cholesterol feature set are provided in Supplementary Table S3. The contextual reference-model comparison for the full five-variable biochemical model is provided in Supplementary Table S4.The screening-oriented threshold sensitivity analysis is provided in Supplementary Table S5. SHAP dependence plots for HbA1c and LDL cholesterol are provided in Supplementary Figures S1 and S2, respectively.

Supplementary Table S1. Algorithm-specific preprocessing, hyperparameter search spaces, and selected hyperparameters used for contextual model benchmarking.

Algorithm	Preprocessing	Hyperparameter search space	Selected hyperparameters
Logistic regression	StandardScaler fitted within training folds	C = 0.01, 0.1, 1, 10, 100; penalty = L2; solver = lbfgs	C = 100; penalty = L2; solver = lbfgs; max_iter = 1000
K-nearest neighbors	StandardScaler fitted within training folds	n_neighbors = 5, 11, 25; weights = uniform, distance; p = 1, 2	n_neighbors = 25; weights = uniform; p = 1
Support vector machine	StandardScaler fitted within training folds	C = 0.1, 1, 10; gamma = scale, auto; kernel = rbf	C = 10; gamma = auto; kernel = rbf; probability = True
Decision tree	No scaling	max_depth = 3, 5, 7, 10, None; min_samples_leaf = 1, 10, 25, 50	max_depth = 7; min_samples_leaf = 50
Random forest	No scaling	n_estimators = 100, 200; max_depth = 5, 10, None; min_samples_leaf = 1, 10, 25; max_features = sqrt	n_estimators = 100; max_depth = None; min_samples_leaf = 25; max_features = sqrt
Gradient boosting	No scaling	n_estimators = 100, 200; learning_rate = 0.05, 0.10; max_depth = 2, 3; min_samples_leaf = 1, 10	n_estimators = 200; learning_rate = 0.10; max_depth = 2; min_samples_leaf = 10
XGBoost	No scaling	n_estimators = 100, 200; learning_rate = 0.05, 0.10, 0.30; max_depth = 2, 3, 6; subsample = 0.8, 1.0; colsample_bytree = 0.8, 1.0	n_estimators = 200; learning_rate = 0.10; max_depth = 2; subsample = 1.0; colsample_bytree = 1.0
Gaussian naïve Bayes	StandardScaler fitted within training folds	var_smoothing = 1e-9, 1e-8, 1e-7	var_smoothing = 1e-9
Multilayer perceptron	StandardScaler fitted within training folds	hidden_layer_sizes = (50,), (100,), (50,50); alpha = 0.0001, 0.001; learning_rate_init = 0.001	hidden_layer_sizes = (50, 50); alpha = 0.001; learning_rate_init = 0.001; max_iter = 200
Spline logistic regression	SplineTransformer + StandardScaler within pipeline	knots = 4; degree = 3; include_bias = False; logistic regression C = 1.0	knots = 4; degree = 3; include_bias = False; C = 1.0; max_iter = 1000

Note: Hyperparameter tuning was performed within the development set using **p**atient-level grouped five-fold cross-validation, ensuring that records from the same patient remained within the same fold**.** The optimal configuration for each algorithm was selected according to the mean cross-validated ROC-AUC. The held-out internal test set was not used during preprocessing, hyperparameter tuning, algorithm selection, or threshold selection. Scaling was applied only to

scale-sensitive algorithms and was fitted within the training folds. This benchmarking analysis was interpreted as a contextual methodological comparison rather than as the primary scientific result of the study. Parameters not listed in the selected configuration retained the corresponding library defaults.

Supplementary Table S2. Leave-one-feature-out analysis for the full five-variable biochemical comparison model.

Removed feature	Accuracy	Sensitivity	Specificity	F1-score	ROC-AUC	Brier score
Glucose	0.876	0.855	0.897	0.874	0.954	0.085
HbA1c	0.845	0.796	0.894	0.838	0.935	0.102
Triglycerides	0.866	0.851	0.881	0.865	0.947	0.091
HDL cholesterol	0.779	0.842	0.716	0.793	0.874	0.144
LDL cholesterol	0.877	0.851	0.902	0.874	0.955	0.085

Note: Each row represents a gradient-boosting model trained after removing one biochemical predictor from the full five-variable biochemical comparison model. The full model included glucose, triglycerides, HDL cholesterol, HbA1c, and LDL cholesterol. Because glucose, triglycerides, and HDL cholesterol are biochemical components of the NCEP ATP III MetS definition, these results were interpreted as secondary analyses of the full comparison model rather than as evidence of independent diagnostic or predictive performance. Threshold-dependent metrics were calculated using the default probability threshold of 0.50. ROC-AUC, receiver operating characteristic area under the curve; HDL, high-density lipoprotein; LDL, low-density lipoprotein; MetS, metabolic syndrome. All models were fitted using the final selected gradient-boosting hyperparameter configuration and evaluated in the patient-level held-out internal test set.

Supplementary Table S3. Patient-clustered logistic regression coefficients for the primary HbA1c–LDL cholesterol feature set.

Predictor	Coefficient	Odds ratio	95% CI for odds ratio	p-value
HbA1c	0.259	1.296	1.250–1.344	<0.001
LDL cholesterol	0.003	1.003	1.002–1.004	<0.001

Note: Logistic regression coefficients were estimated as a post-evaluation descriptive analysis using the primary HbA1c–LDL cholesterol feature set, with patient-clustered robust standard errors to account for repeated records from the same patient. This analysis was not used for hyperparameter tuning, algorithm selection, threshold selection, final model training, or held-out test-set performance estimation. Coefficients are unstandardized and represent the change in log-odds of reference-defined MetS per one-unit increase in each predictor. Odds ratios are reported per one-unit increase. CI, confidence interval; LDL, low-density lipoprotein; MetS, metabolic syndrome.

Supplementary Table S4. Contextual reference-model comparison for the full five-variable biochemical model in the patient-level held-out internal test set.

Model	Accuracy	Sensitivity	Specificity	F1	ROC-AUC (95% CI)	Brier
Logistic Regression	0.620	0.522	0.719	0.580	0.670 (0.654–0.685)	0.232
Spline Logistic Regression	0.675	0.655	0.695	0.669	0.721 (0.707–0.733)	0.215
Gradient Boosting	0.879	0.865	0.894	0.878	0.956 (0.950–0.961)	0.084

Note: This supplementary table presents a contextual comparison of the full five-variable biochemical model with standard logistic regression, spline logistic regression, and gradient boosting in the held-out internal test set. The full model included glucose, triglycerides, HDL cholesterol, HbA1c, and LDL cholesterol. Because glucose, triglycerides, and HDL cholesterol are biochemical components of the NCEP ATP III MetS definition, these results were interpreted as secondary contextual comparisons rather than as independent diagnostic or predictive performance. ROC-AUC, receiver operating characteristic area under the curve; CI, confidence interval; MetS, metabolic syndrome; HDL, high-density lipoprotein; LDL, low-density lipoprotein. All models were fitted using the development set and evaluated in the patient-level held-out internal test set. ROC-AUC confidence intervals were estimated using patient-level bootstrap resampling with 1000 iterations.

Supplementary Table S5. Screening-oriented threshold sensitivity analysis for the primary HbA1c–LDL cholesterol gradient-boosting model.

Threshold	Sensitivity	Specificity	PPV	NPV	Accuracy	FP	FN
0.500	0.824	0.641	0.698	0.783	0.733	960	475
0.400	0.901	0.548	0.668	0.846	0.725	1,208	267

Note: The screening-oriented threshold was derived from out-of-fold predictions obtained by patient-level grouped five-fold cross-validation within the development set to achieve approximately 90% sensitivity. This fixed threshold was then applied to the patient-level held-out internal test set without further optimization. Threshold-dependent performance metrics are reported for comparison with the default 0.50 threshold. PPV, positive predictive value; NPV, negative predictive value.

Supplementary Figure S1. SHAP dependence plot for HbA1c in the patient-level held-out internal test set.

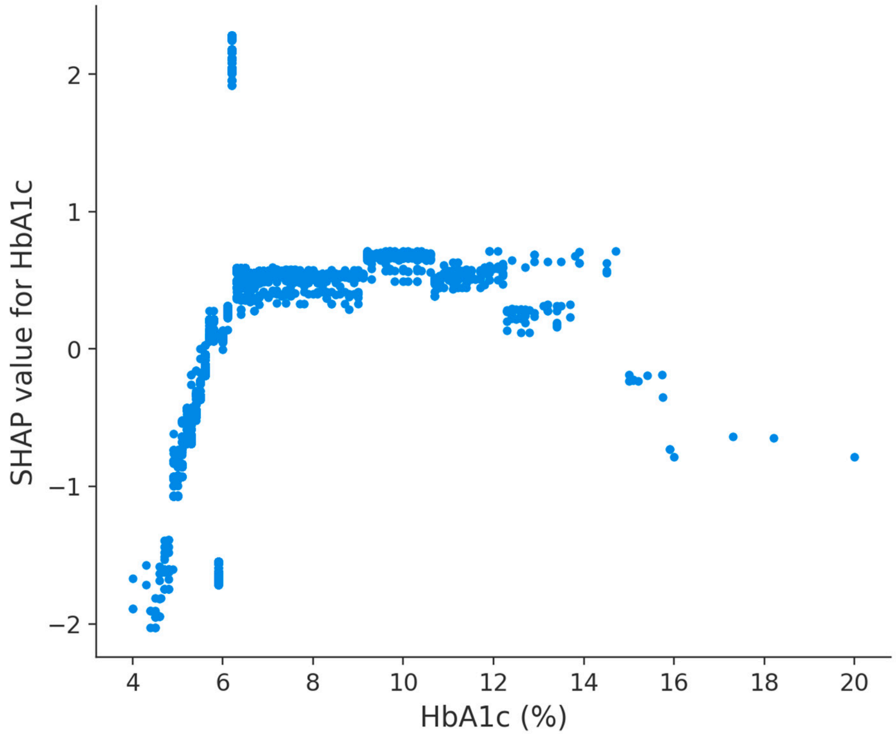

Supplementary Figure S2. SHAP dependence plot for LDL cholesterol in the patient-level held-out internal test set.

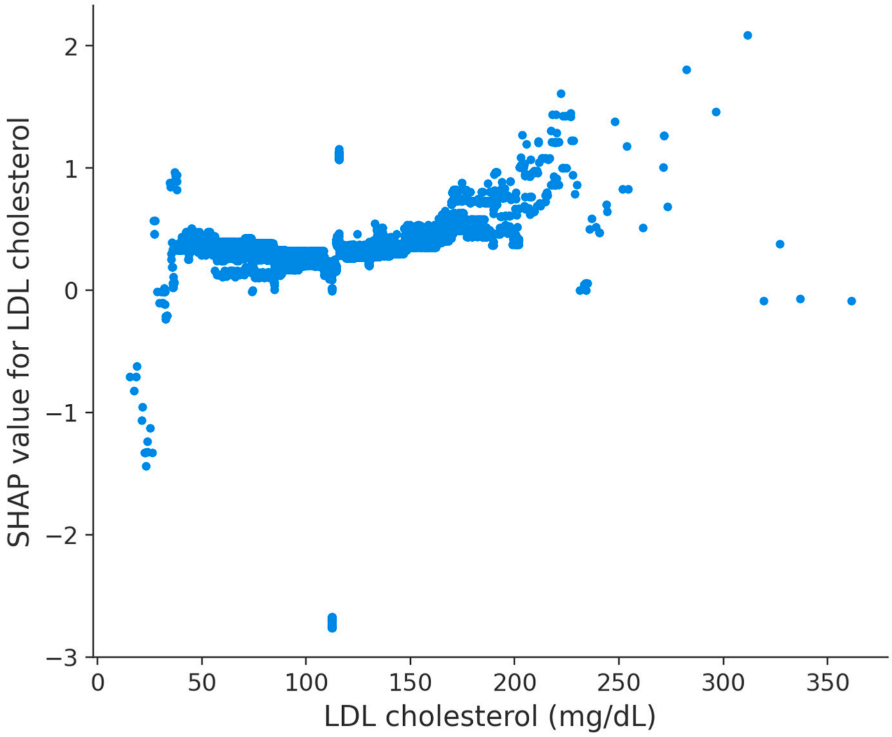

Supplement: Supplementary file 1 [file diagnostics-16-03048-s001.zip › Supplementary Materials.pdf]
