# Supplementary material for: Screening Signals of Reference-Defined Metabolic Syndrome Using HbA1c and LDL Cholesterol: An Explainable Machine Learning Study
Source: Diagnostics (Basel). 2026 Sep 20;16(18):3048. doi: 10.3390/diagnostics16183048 (PMC13605401; doi:10.3390/diagnostics16183048)
Supplement: Supplementary file 1 [file diagnostics-16-03048-s001.zip › Supplementary_File_S1_TRIPOD_AI_Checklist_UPDATED.pdf]

Supplementary File S1
TRIPOD+AI Reporting Checklist

Manuscript: "Screening Signals of Reference-Defined Metabolic Syndrome Using HbA1c and LDL Cholesterol: An Explainable Machine Learning Study"

This checklist was completed using the TRIPOD+AI 2024 reporting guideline for studies developing or evaluating clinical prediction models using regression or machine-learning methods. D = model development; E = model evaluation. Page/section references correspond to the current revised manuscript and should be updated if pagination changes before submission. Source guideline: Collins GS, Moons KGM, Dhiman P, et al. BMJ. 2024;385:e078378.

Section/Topic	Item	D/E	TRIPOD+AI checklist item	Reported on page/section	Status
TITLE	1	D;E	Identify the study as developing or evaluating a multivariable prediction model, the target population, and the outcome.	p. 1 (Title)	Reported
ABSTRACT	2	D;E	Provide a structured abstract addressing the TRIPOD+AI for Abstracts items.	pp. 1–2 (Abstract)	Reported
INTRODUCTION	3a	D;E	Explain the healthcare context and rationale, including references to existing models.	pp. 2–4 (Introduction)	Reported
INTRODUCTION	3b	D;E	Describe the target population, intended purpose in the care pathway, and intended users.	§1 (Introduction, study aim/final paragraphs); §2.7; §4 (clinical workflow discussion)	Partially reported
INTRODUCTION	3c	D;E	Describe known health inequalities between sociodemographic groups.	Not reported	Not reported
INTRODUCTION	4	D;E	Specify the study objectives and whether model development, validation/evaluation, or both were undertaken.	p. 4 (Introduction, study aim)	Reported
METHODS—Data	5a	D;E	Describe data sources separately for development and evaluation, rationale for using the data, and representativeness.	§2.1; §2.5 (data source, record flow, curation, and representativeness caveat)	Reported
METHODS—Data	5b	D;E	Specify dates of participant data collection and, if applicable, follow-up.	p. 5 (§2.1)	Reported
METHODS—Participants	6a	D;E	Specify the study setting, number of centres, and geographical location.	p. 5 (§2.1)	Reported
METHODS—Participants	6b	D;E	Describe eligibility criteria.	p. 5 (§2.1)	Reported
METHODS—Participants	6c	D;E	Describe relevant treatments and how they were handled.	Not applicable (screening-oriented retrospective modelling study; no intervention)	N/A
METHODS—Data preparation	7	D;E	Describe data preprocessing and quality checking, including consistency across sociodemographic groups.	§§2.5–2.6; Supplementary Table S1	Partially reported
METHODS—Outcome	8a	D;E	Clearly define the predicted outcome and time horizon, how and when it was assessed, and rationale.	pp. 5–6 (§2.3)	Reported
METHODS—Outcome	8b	D;E	If outcome assessment requires subjective interpretation, describe assessor qualifications and demographics.	Not applicable (criteria-based outcome definition)	N/A
METHODS—Outcome	8c	D;E	Report actions to blind outcome assessment.	Not applicable/Not reported; outcome derived from existing criteria-based records	N/A
METHODS—Predictors	9a	D	Describe the choice and any preselection of initial predictors.	§1 (rationale); §2.4 (prespecified feature sets)	Reported
METHODS—Predictors	9b	D;E	Clearly define predictors, including how and when measured, units, and any blinding.	§§2.1, 2.3–2.4; Table 1	Partially reported

Section/Topic	Item	D/E	TRIPOD+AI checklist item	Reported on page/section	Status
METHODS—Predictors	9c	D;E	If predictor measurement is subjective, describe assessor qualifications and demographics.	Not applicable (objective laboratory measurements)	N/A
METHODS—Sample size	10	D;E	Explain how the study size was determined and justify sufficiency, including any calculation.	Not reported	Not reported
METHODS—Missing data	11	D;E	Describe how missing data were handled and reasons for omitting data.	pp. 5–6 (§§2.1, 2.5)	Reported
METHODS—Analytical methods	12a	D	Describe how data were used and partitioned, considering sample-size requirements and leakage.	p. 6 (§2.5)	Reported
METHODS—Analytical methods	12b	D	Describe predictor handling, including functional form, scaling, transformations, or standardisation.	pp. 6–7 (§§2.5–2.6; Supplementary Table S1)	Reported
METHODS—Analytical methods	12c	D	Specify model types, rationale, all model-building steps, hyperparameters, and internal validation.	pp. 7–8 (§§2.6–2.9; Supplementary Table S1)	Reported
METHODS—Analytical methods	12d	D;E	Describe handling and quantification of heterogeneity across clusters.	Not applicable (single-centre dataset)	N/A
METHODS—Analytical methods	12e	D;E	Specify all performance measures and plots, with rationale, used to evaluate and compare models.	pp. 7–8 (§2.7)	Reported
METHODS—Analytical methods	12f	E	Describe any model updating or recalibration arising from evaluation.	Not applicable (no model updating performed)	N/A
METHODS—Analytical methods	12g	E	For evaluation of an existing model, describe how predictions were calculated.	Not applicable (new models developed in this study)	N/A
METHODS—Class imbalance	13	D;E	If class-imbalance methods were used, explain why and how, including recalibration.	p. 6 (§2.5; no resampling used)	Reported
METHODS—Fairness	14	D;E	Describe approaches used to address model fairness and their rationale.	Not assessed; age and sex were available descriptively but were not included as model predictors or used for formal fairness analysis	Not reported
METHODS—Model output	15	D	Specify model output and rationale for classification thresholds.	§2.7; §3.6; Table 5; Supplementary Table S5	Reported
METHODS—Training vs evaluation	16	D;E	Identify differences between development and evaluation data in setting, eligibility, outcome, and predictors.	p. 6 (§2.5; random split from the same dataset)	Reported
METHODS—Ethical approval	17	D;E	Name the ethics committee and describe informed consent or waiver.	p. 5 (§2.2); p. 23 (back matter)	Reported
OPEN SCIENCE—Funding	18a	D;E	Give funding source and funder role.	p. 23 (Funding)	Reported
OPEN SCIENCE—Conflicts	18b	D;E	Declare conflicts of interest and financial disclosures.	p. 23 (Conflicts of Interest)	Reported
OPEN SCIENCE—Protocol	18c	D;E	State where the protocol can be accessed or that no protocol was prepared.	Not reported	Not reported
OPEN SCIENCE—Registration	18d	D;E	Provide registration information or state that the study was not registered.	Not reported	Not reported
OPEN SCIENCE—Data sharing	18e	D;E	Provide details of study-data availability.	p. 23 (Data Availability Statement)	Reported
OPEN SCIENCE—Code sharing	18f	D;E	Provide details of analytical-code availability.	Not reported	Not reported
PATIENT/PUBLIC INVOLVEMENT	19	D;E	Report patient and public involvement or state that there was none.	Not reported	Not reported

Section/Topic	Item	D/E	TRIPOD+AI checklist item	Reported on page/section	Status
RESULTS—Participants	20a	D;E	Describe participant/record flow and numbers with and without the outcome; diagram if helpful.	pp. 1–2 (Abstract); pp. 8–9 (§3.1)	Reported
RESULTS—Participants	20b	D;E	Report participant characteristics, key predictors, dates, sample size, outcome events, follow-up, and missing data.	§3.1; Table 1; §§2.1–2.3 (sample size, outcome completeness, dates, and missing-data exclusions)	Reported
RESULTS—Participants	20c	E	For model evaluation, compare important predictor distributions with development data.	Not applicable/Not reported; evaluation used a random split from the same source population	N/A
RESULTS—Model development	21	D;E	Specify numbers of participants/records and outcome events in each analysis.	pp. 1–2, 6, 10–11 (Abstract; §§2.5, 3.3–3.4)	Reported
RESULTS—Model specification	22	D	Provide the full model or implementation resource to enable third-party prediction and evaluation.	pp. 21–22 (Supplementary Table S1); full code/model object not provided	Partially reported
RESULTS—Model performance	23a	D;E	Report performance estimates with confidence intervals, including key subgroups; use plots as appropriate.	§§3.2–3.6; Tables 2, 4–5; Figures 2–4; Supplementary Table S5	Reported
RESULTS—Model performance	23b	D;E	If examined, report heterogeneity in model performance across clusters.	Not applicable (single-centre; no cluster analysis)	N/A
RESULTS—Model updating	24	E	Report results of model updating and subsequent performance.	Not applicable (no updating performed)	N/A
DISCUSSION—Interpretation	25	D;E	Provide an overall interpretation in context of objectives, prior studies, and fairness; avoid overinterpretation.	pp. 18–20 (§4 Discussion)	Reported
DISCUSSION—Limitations	26	D;E	Discuss limitations and effects on bias, uncertainty, and generalisability.	§4 (Limitations: single-center design, repeated records/within-patient dependence, residual incorporation bias, prevalence distortion, and external validation)	Reported
DISCUSSION—Usability	27a	D	Describe how unavailable or poor-quality inputs should be handled during implementation.	Not reported	Not reported
DISCUSSION—Usability	27b	D	Describe user interaction and expertise/training needed to use the model.	Not reported	Not reported
DISCUSSION—Usability	27c	D;E	Discuss next research steps regarding applicability and generalisability.	§4 (Discussion); §5 (Conclusions: external, temporal, multicenter, and prospective validation)	Reported
